# Cancer stem cells as targets for DC-based immunotherapy of colorectal cancer

**DOI:** 10.1038/s41598-018-30525-3

**Published:** 2018-08-13

**Authors:** Magdalena Szaryńska, Agata Olejniczak, Jarosław Kobiela, Dariusz Łaski, Zbigniew Śledziński, Zbigniew Kmieć

**Affiliations:** 1 Department of Histology, Medical University of Gdansk, 80-210 Gdansk, Poland; 2 Department of General, Endocrine and Transplant Surgery, Medical University of Gdansk, 80-214 Gdansk, Poland

## Abstract

The therapy of colorectal cancer (CRC) patients is often unsuccessful because of the presence of cancer stem cells (CSCs) resistant to conventional approaches. Dendritic cells (DC)-based protocols are believed to effectively supplement CRC therapy. Our study was aimed to assess how the number and properties of CSCs isolated from tumor tissue of CRC patients will affect the biological characteristics of *in vitro* modified DCs. Similar procedures were conducted with the using of CRC HCT116 and HT29 cell lines. We found that the detailed configuration of CSC-like markers significantly influenced the maturation and activation of DCs after stimulation with cancer cells lysates or culture supernatants. This basic stimulatory effect was enhanced by LPS that is normally present in CRC CSCs niche. The increased number of CD29^+^ and CD44^+^ CSCs presented the opposite impact on treated DCs as showed by many significant correlations. The CD133^+^ CSCs seemed to impair the functions of DCs. The more CD133^+^ CSCs in tumor sample the lower number of activated DCs evidenced after stimulation. Moreover, our results showed superiority of the spherical culture model over the adherent one since spherical HCT116 and HT29 cells presented similar influence on DCs properties as CRC patients cancer cells. We concluded that the DCs features may depend directly on the properties of CSCs affected by progression status of tumor.

## Introduction

Colorectal cancer (CRC) is one of the most frequent malignancies and the fourth most common cause of cancer-related deaths in the world with 1.2 million new cases being diagnosed every year. The 5-year survival rate of patients with stage IV CRC is less than 10%^1,2^. Despite increasing knowledge concerning pathogenesis, genetic and epigenetic alterations associated with the CRC development, effectiveness of the therapy remains unsatisfactory because of cancer recurrence and metastases. Cancer stem cells (CSCs) were showed to be responsible for metastasis, recurrence, relapse and resistance to conventional chemotherapy^3,4^ which can destroy only proliferating and mature cancer cells while quiescent CSCs survive. Therefore, elucidation of the mechanisms of CSCs maintenance is important for the understanding of cancer cell persistence and relapses. Additionally, that may enable specific CSCs targeting as a potential therapeutic strategy to definitively eradicate cancer^5–7^. The CSC-specific immune responses in breast cancer and glioblastoma^8–10^ were proved; despite the immune evasion of the CSCs. Vaccination of dendritic cells (DCs) with irradiated glioma tumorspheres was demonstrated to increase the survival rate in a mice cancer model^9^.

The main goal of current efforts worldwide is to incorporate recent discoveries into novel treatment algorithms. One of the potential methods is immunotherapy which is hoped to induce CRC-specific cytotoxic reactions mediated by antigen presenting cells (APCs) (including DCs), helper CD4^+^ and effector CD8^+^ T lymphocytes^11,12^. Although many tumor-associated antigens (TAA) have been already found in CRC cells, such as CEA (carcinoembryonic antigen)^13,14^, WT1 (Wilms’ tumor gene 1)^15,16^, MUC1 (mucin 1)^13^, MAGE (melanoma-associated antigen gene)^17–19^, p53^20^, the heterogeneity and patient-specificity are severe obstacles to use the anti-TAA therapies. A very promising strategy to prime cancer-specific T cell responses is dendritic cell-based immunotherapy. Autologous cancer cells lysates could provide a wide range of ‘personalized’ cancer epitopes including neoantigens which result from cancer–specific DNA mutations^21^. Cancer immunotherapy approaches based on the vaccination with the use of TAA, whole cancer cells or viral vectors, have been tested to treat CRC patients. However, despite the relative effectiveness of these treatments side-effects are still observed in the large proportion of patients and the number of recurrences is still high^13,16,17,20,22–25^.

Dendritic cells qualitatively and quantitatively coordinate the function of the immune system cells such as various populations of T lymphocytes, also naïve and memory B cells, natural killer (NK) cells and NKT cells through the secretion of cytokines (IL-10, IL-12, IL-15, IFNs) or the presence in their cell membranes various proteins such as CD1, CD54, CD80, CD83, CD86, CCR7^26–28^. The main role of DCs is to mediate innate immune responses and induce adaptive responses acting as powerful APCs^29^. DCs represent a widely distributed heterogeneous population of professional APCs that originate from bone marrow precursors known as MDPs (monocyte and DC progenitors)^30^. The critical issues underlying DC-immunotherapy is limited number of DCs available from each patient and, additionally, those DCs can represent variable activities: antigen presenting, cytotoxic^31,32^ or suppressive^33–35^.

It was reported that spheroid (3-D) cultures of cancer cell lines better than adherent (2-D) cell cultures resemble original cancer in such areas as gene expression profiles, cellular heterogeneity, morphology and distribution of cancer cells^36–42^. These aspects of cancer cells biology primarily depend on the access to oxygen, nutrients and growth factors. Studies conducted on tumorospheres derived from various types of cancers, including breast^43–45^, colon^11,12,37,39,46^, lung^47,48^ and prostate^49^ cancer as well as glioma^8^ and melanoma^50^ showed that sphere-based assays could be a reliable platform for development of immunotherapy targeting CSCs. It is also believed that spherical cultures can provide short-term patient-derived CSCs for the evaluation of DC-based therapies what defined the main goals of our study.

Cancer cells expanded in the spheroid form were showed to induce different immune response in comparison to cells cultured in adherent form^3^, since they were found to present lower levels of TAA and MHC class I proteins^3^. Additionally, it was showed that spherical cell cultures can secrete IL-4 that inhibited functions of cytotoxic T lymphocytes and NK cells^51–53^.

The discovery of cancer stem cells enables the understanding of some of the most lethal features of cancer. The CSC model states that cells within the tumor are hierarchically organized with the subpopulation of cancer stem cells with high tumorigenicity on the top. CSCs were showed to be able to self-renew and to generate differentiated cells within the tumor mass often described as non-CSCs^54,55^. These cells are also responsible for the main clinical complications of anti-cancer therapies including appearance of relapses, sometimes years after chemo- or radiotherapy^56^. Therefore, elucidating the mechanisms of CSC maintenance is important for our understanding of tumor cells persistence and relapses and may enable specific targeting of CSCs, a promising therapeutic strategy to eradicate cancer^57,58^. The identification and classification of CSCs remains controversial, as none of the known markers are universal and reliable for the identification of CSCs in all types of tumors^59^. The most commonly used marker of CSCs (including CRC) is CD133 protein (prominin-1)^59^. According to some analyses only CD133^+^ cells are able to reproduce a CRC tumor in a mouse xenotransplantation model^4,60^. However, some other groups showed independently that CD133^−^ cells also possess high proliferative and differentiating potential, comparable to those of CD133^+^ CRC-CSCs^4,61^. Proteins which are also considered as reliable CSCs markers are CD24, CD29, CD44, CD166, Aldh1 and other^36,37,62,63^.

The analyses of the expression profiles of co-stimulatory molecules and cytokines suggest that CSCs might contribute to the formation of an immune‐suppressive cancer microenvironment including immune escape from cytotoxic T lymphocytes (CTLs)^51,53^. Although CSCs could inhibit differentiation of CD8^+^ naïve T cells, they could not inhibit the cytotoxicity of previously differentiated and activated CTLs^51,53^. Vaccination of CRC patients with autologous, cancer lysate-loaded DCs has been showed recently to be a well-tolerated therapeutic approach^64,65^ but the role of CSCs has not yet been analyzed.

The aim of our study was to evaluate how the CSC-like properties may influence the effectiveness of DC stimulation and modulate their phenotype, endocytotic activity and ability to induce the proliferation of lymphocytes. According to our best knowledge no other study has addressed the evaluation of specific DCs pulsed with CSC-antigens-rich lysates and tumor conditioned medium (TCM) activities in such an ample repertoire of *in vitro* experiments. We hoped to provide the valuable information which could contribute to the clinical applications of such therapies.

## Results

### The establishment of primary spherical cultures of colorectal cancer cells and expansion of cancer stem cells

We collected fresh CRC specimens from 27 patients who had not received chemo- or radiotherapy prior to surgery. It was possible to establish spherical cultures from the cancer tissue of 18 patients, however, only five could be maintained in culture for extended passages. We used these primary spherical cultures up to ten passages for our analysis. In the remaining cultures cancer cells did not survive *ex vivo* or lost their sphere-forming capacity after first passages. At the same time we wanted to highlight the fact that our functional tests were conducted with cells, TCM or lysates obtained from third passage, although the phenotypic analyses showed that CSCs along the expansion were stable what is presented in supplementary figures (Figs S1, S2, S3).

The additional clinicopathological information concerning CRC patients included into the study who represent the group of donors which CSCs survived in culture for extended passages are presented in Supplementary Table S1. Moreover, we presented the gating strategy for CSCs obtained from tumor fragments of patients in Supplementary Fig. S4.

We identified significant correlation between TNM status and the life span in culture of CSCs derived from CRC patients (R = 0.65, *p* < *0*.*05*). The cancer tissue samples which gave the most stable spherical cultures were collected from four patients in stage III and one patient in stage IV of CRC (according to the current TNM staging). Moreover, the presence of lymph node metastasis correlated with the proportion of CD133^+^CD44^−^CD29^+^ CSCs during expansion (R = 0.52, *p* < *0*.*05*).

### Impact of TNM status on DCs derived from CRC patient-derived monocytes

**Figure 1:**
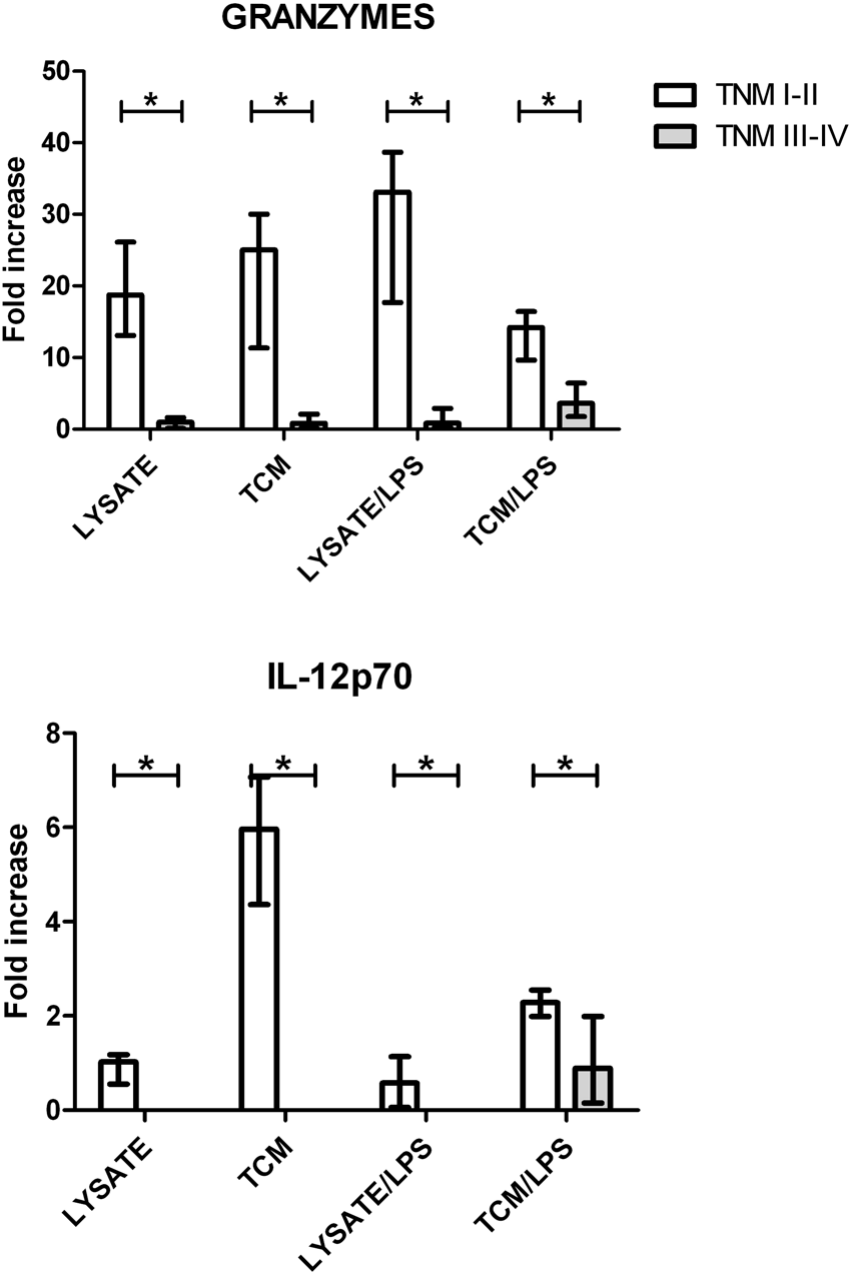
The fold increase of granzymes and IL-12p70 concentrations in supernatants collected from *in vitro* modified DCs derived from colorectal cancer (CRC) patients blood monocytes. Autologous cancer cells lysates and tumor conditioned media (TCM) used in these analyses were collected from spherical cultures of CRC patients-derived CSCs (cancer stem cells). Bars and whiskers represent mean ± SEM (*p < *0*.*05* TNM I-II vs TNM III-IV, ANOVA Kruskal – Wallis test, n = 4–6).

Afterwards, we analyzed the impact of CRC TNM status on some biological features of DCs derived from monocytes of CRC patients blood. We revealed significant correlation between TNM status and the concentrations of granzymes (R = 0.61, *p* < *0*.*05*) and IL12-p70 (R = 0.79, *p* < *0*.*05*) in the media from DCs culture. The substantially higher concentrations of IL-12p70 and granzymes in the supernatants from DCs cultures were found in samples which originated from CRC patients with TNM I-II stages in comparison to patients with more advanced stage (TNM III-IV). The same relationships were observed in all applied options of the DCs stimulation (Fig. 1). The analysis of influence of TNM progression status on other CRC patient–derived DCs features as phenotype, endocytotic and cytotoxic activities did not reveal any significant differences.

### The morphology of dendritic cells in culture

**Figure 2:**
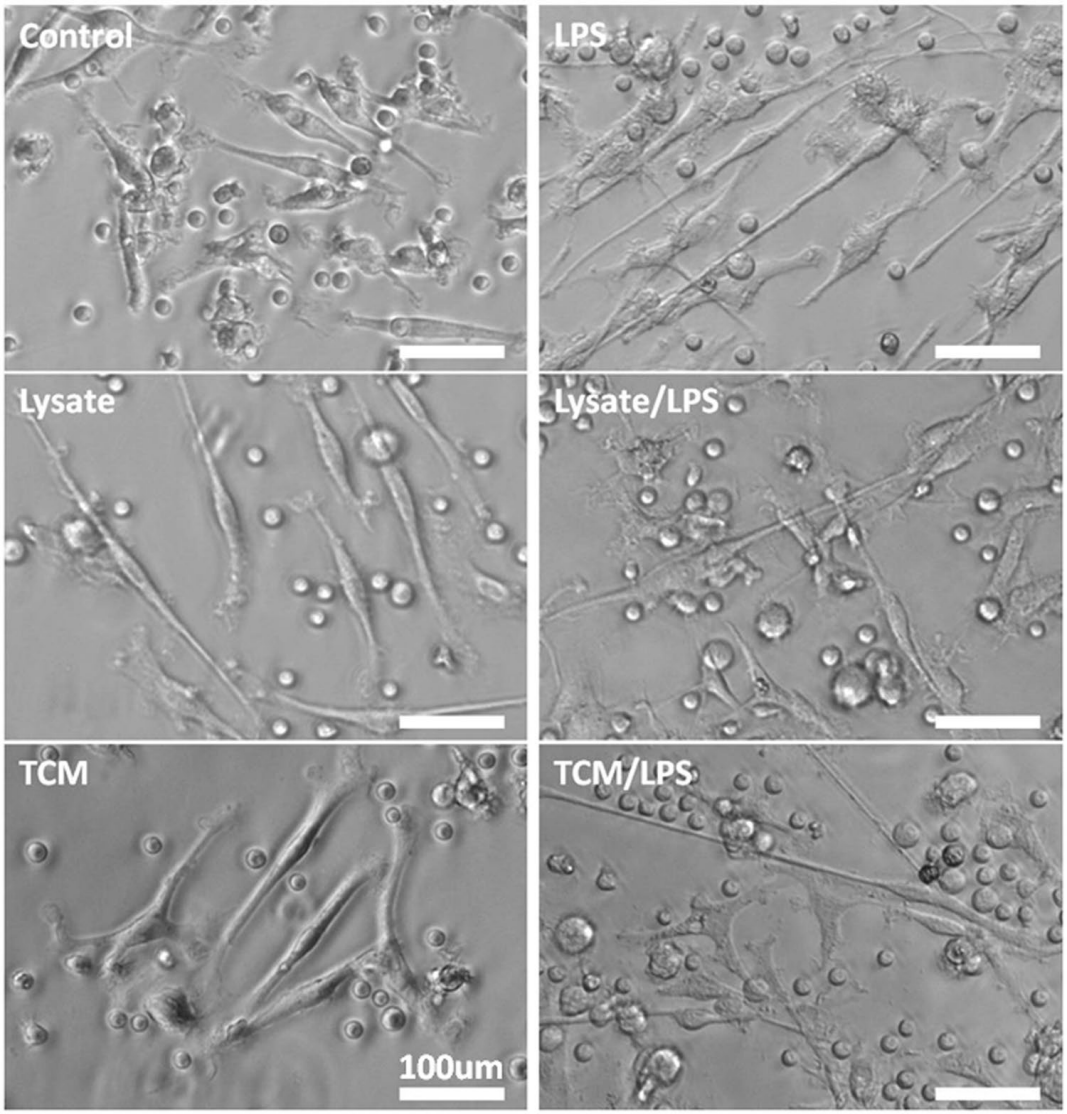
The morphology of DCs expanded from monocytes and incubated for 24 h with allogenic lysates, TCM, LPS or with mixtures of lysates and TCM with LPS in comparison to control immature DCs (iDCs). Scale bars represent 100 µm.

**Figure 3:**
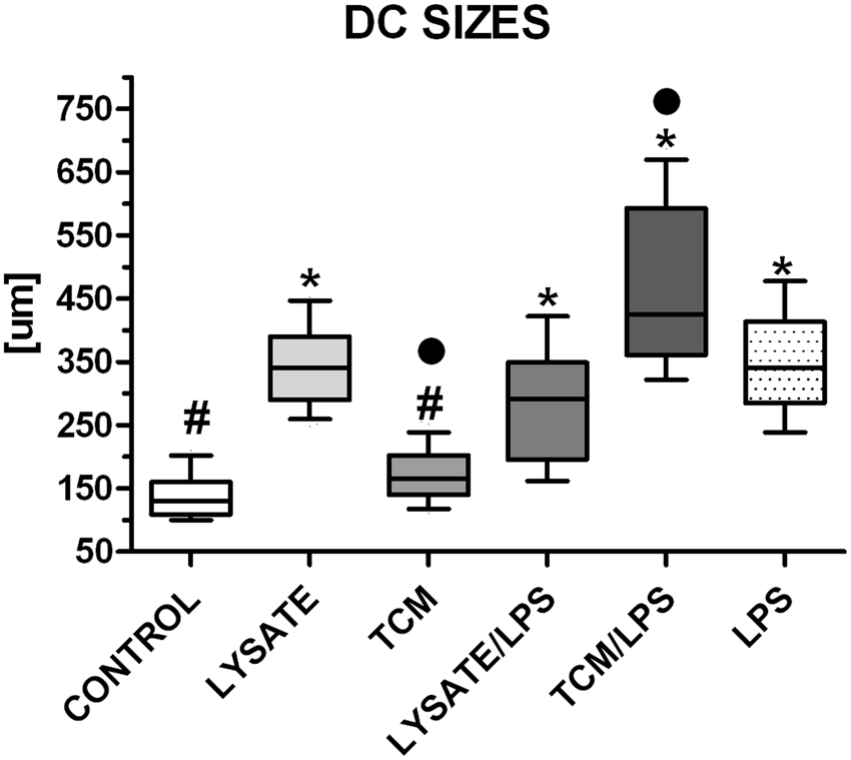
The sizes [µm] of DCs expanded from monocytes and incubated for 24 h with lysates, TCM, LPS or with mixtures of lysates and TCM with LPS in comparison to iDCs. Statistical significance was measured in comparison to iDCs. Bars and whiskers represent mean ± SEM (*p < 0.05 vs Control, ^•^*p* < 0.05 TCM vs TCM/LPS, ^#^*p* < 0.05 vs LPS; ANOVA Kruskal – Wallis test, n = 25).

We used the same procedure for establishing DC cultures from their monocytic precursors from the blood of both types of donors: healthy subjects and CRC patients. Six days after our cultures started, the cells displayed fibroblast–like morphology with fine cytoplasmic processes, typical for DCs morphology (Fig. 2). The stimulation for 24 h with lysates of cancer cells, TCM, LPS or the combinations of these agents resulted in longer processes of DCs, however, this effect depended on the type of used agent (Fig. 3). The incubation with TCM/LPS resulted in the cells with the most extended morphology (up to 780 µm), even more relevant than observed after stimulation with LPS alone, although the TCM alone did not affect length of cell processes when compared to immature DCs (iDCs). After incubation with lysate/LPS and TCM/LPS for 24 h the vast majority of cells showed flat cellular forms and adhered to culture dishes. The similar features were observed for DCs originating from blood of healthy volunteers and CRC patients.

### The phenotype of dendritic cells isolated from blood of CRC patients and healthy subjects

**Figure 4:**
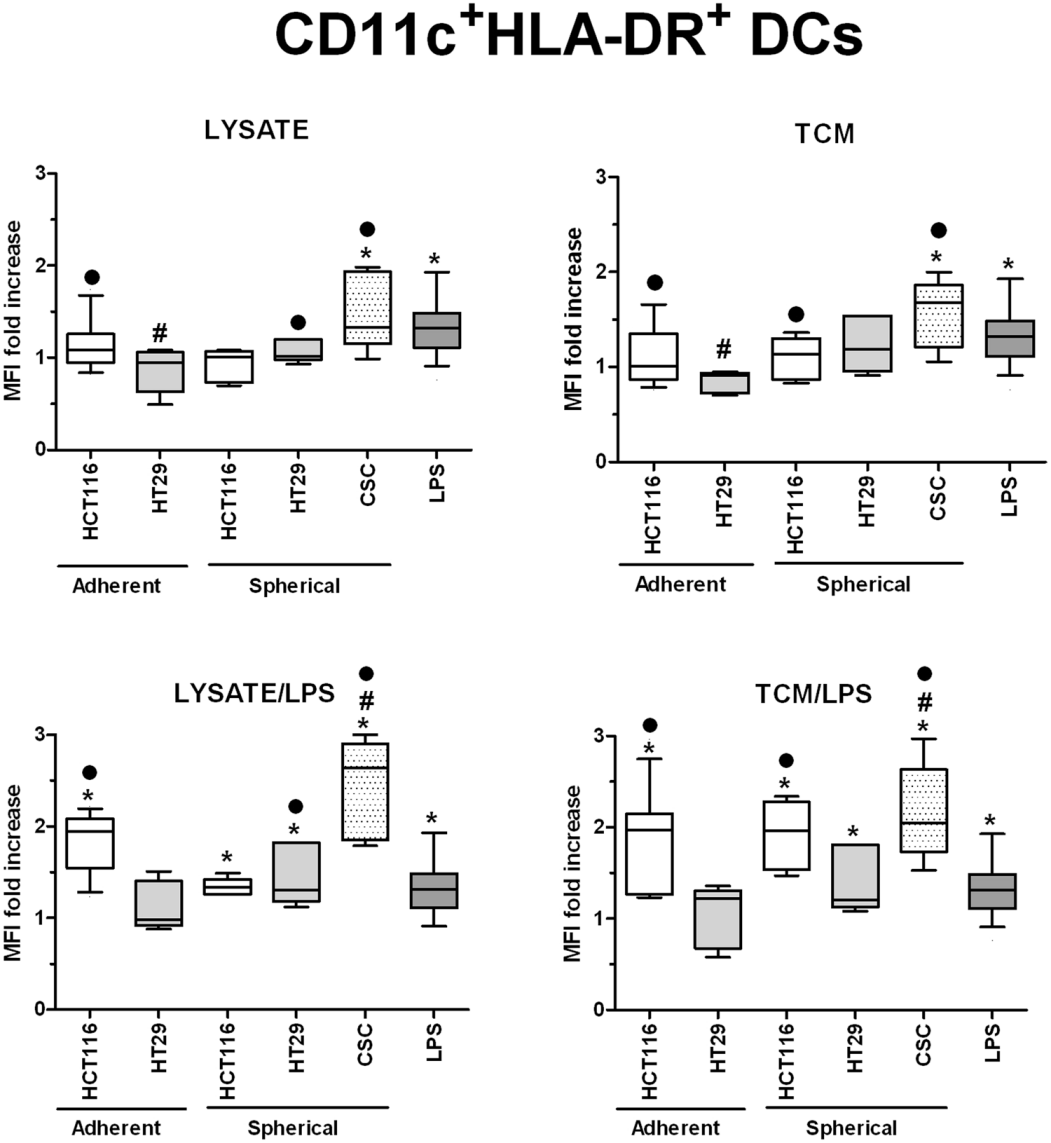
The effect of cancer cell lysates, TCM and LPS on the proportions of CD11c^+^HLA-DR^+^ DCs. Data presented as MFI fold change over the level obtained from iDCs culture. Lysates and TCM used in these analyses were collected from cultures of HCT116 and HT29 CRC lines expanded in adherent or spherical forms (allogenic), and CRC patients-derived CSCs (autologous). The control value of iDCs was set at the level of 1. Bars and whiskers represent mean ± SEM (*p < *0*.*05* vs iDCs, ^•^*p* < *0*.*05* lysate vs lysate/LPS or TCM vs TCM/LPS, ^#^*p* < *0*.*05* vs LPS; ANOVA Kruskal – Wallis test, n = 10–15).

**Figure 5:**
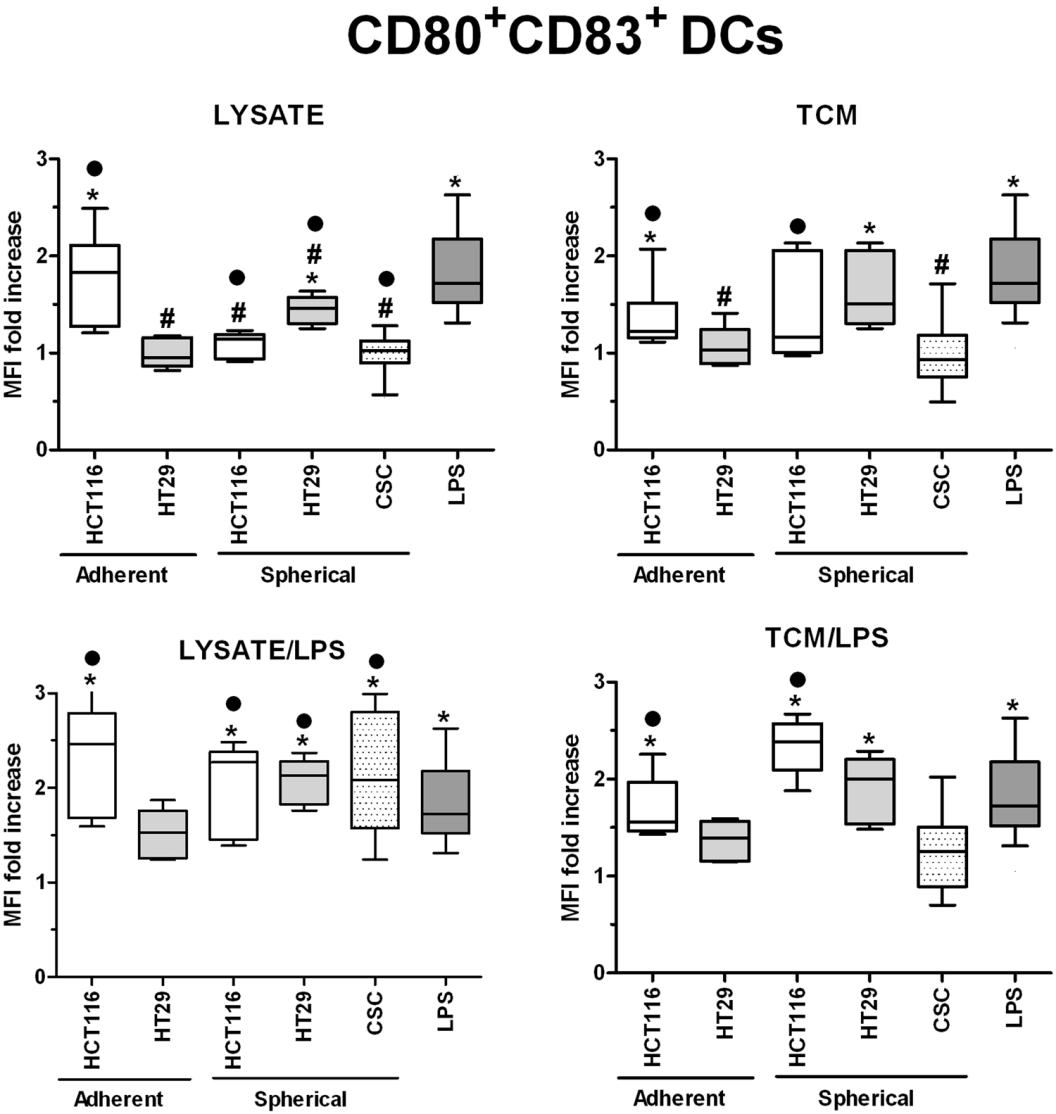
The effect of cancer cell lysates, TCM and LPS on the proportions of CD80^+^CD83^+^ DCs. Data presented as MFI fold change over the level obtained from iDCs culture. Lysates and TCM used in these analyses were collected from cultures of HCT116 and HT29 CRC lines expanded in adherent or spherical forms (allogenic), and CRC patients-derived CSCs (autologous). The control value of iDCs was set at the level of 1. Bars and whiskers represent mean ± SEM (*p < *0*.*05* vs iDCs, ^•^*p* < *0*.*05* lysate vs lysate/LPS or TCM vs TCM/LPS, ^#^*p* < *0*.*05* vs LPS; ANOVA Kruskal – Wallis test, n = 10–15).

**Figure 6:**
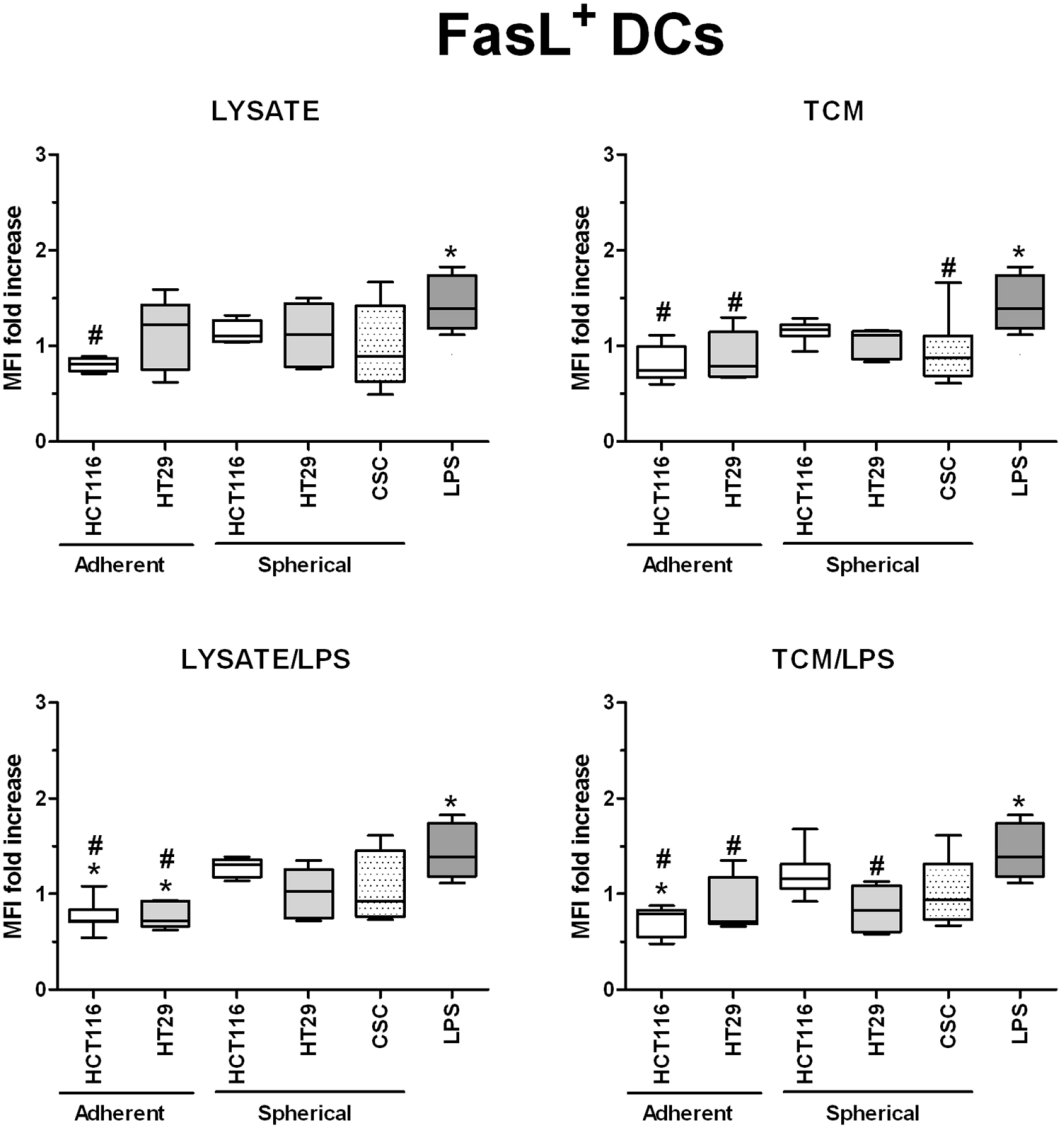
The effect of cancer cell lysates, TCM and LPS on the proportions of FasL^+^ DCs. Data presented as MFI fold change over the level obtained from iDCs culture. Lysates and TCM used in these analyses were collected from cultures of HCT116 and HT29 CRC lines expanded in adherent or spherical forms (allogenic), and CRC patients-derived CSCs (autologous). The control value of iDCs was set at the level of 1. Bars and whiskers represent mean ± SEM (*p < *0*.*05* vs iDCs, ^#^*p* < *0*.*05* vs LPS; ANOVA Kruskal – Wallis test, n = 10–15).

In order to investigate in details the phenotypic differences between DCs obtained during diverse experimental procedures we analyzed cytometricaly DCs gained during the differentiation of monocytic precursors from peripheral blood of both healthy volunteers and CRC patients. The gating strategy is presented in Supplementary Fig. S5. We found that the use of various stimulatory agents differently affected the proportions of CD11c^+^HLA-DR^+^ (Fig. 4), CD80^+^CD83^+^ (Fig. 5) and FasL^+^ (Fig. 6) DCs. LPS, a potent DC activator which was used as the specific internal positive control of stimulatory potential, triggered a significant maturation of all DC populations. The incubation with lysate/LPS or TCM/LPS most efficiently increased the number of mature and activated DCs. The statistically significant differences were the most relevant especially for CD80^+^CD83^+^ DCs.

The lysates and TCM of CSCs from CRC patients increased the proportion of CD11c^+^HLA-DR^+^ DCs significantly higher in comparison to DCs exposed to lysates and TCM from HCT116 and HT29 cell cultures (Fig. 4) and even higher than DCs incubated in the presence of LPS alone. The proportion of CD11c^+^HLA-DR^+^ cells stimulated with both of our agents (±LPS) increased equally.

CSC-derived lysates in the combination with LPS elevated the CD80^+^CD83^+^ DCs proportion in the most significant manner and it is even more meaningful as lysates alone lowered the MFI of CD80^+^CD83^+^ cells below the LPS-stimulated DCs level. At the same time, TCM exerted much weaker influence since we observed the proportion of CD80^+^CD83^+^ DCs at the level of iDCs and significantly lower than LPS-stimulated DCs (Fig. 5). The analysis of differences in the proportions [%] of DCs with particular phenotype presented similar tendencies but more pronounced what strongly confirmed our observations.

Because FasL can induce apoptosis of the cells with appropriate receptor on the surface, including cancer cells and immune effectors (especially cytotoxic T cells)^66^ we decided to analyze how lysates or TCM (with or without LPS) would affect the number of FasL^+^ DCs. We found that factors derived from adherent cultures of HCT116 and HT29 cells significantly decreased the proportion of FasL^+^ DCs in comparison to iDCs (Fig. 6), what suggested that these samples exerted the inhibitory effect referring to this DC feature. The most of our samples didn’t influence the FasL level on DCs since the proportion of FasL^+^ cells was fixed on the same level as iDCs and at the same time, lower than LPS-stimulated DCs.

Interestingly, the presence of some specific markers on the CSCs surface was found to influence the effectiveness of DCs maturation and activation after the stimulatory procedure while using lysates or TCM. We presented in Table 2 numerous significant correlations between the frequencies of CD11c^+^HLA-DR^+^and CD80^+^CD83^+^ DCs and the proportions of CSCs with specific phenotype. The negative correlations between CD11c^+^HLA-DR^+^ DCs and the presence of CD44^+^CD29^−^ CSCs in both CD133^+^ and CD133^−^ fractions indicated the negative impact of CD44^+^ CSCs on the *in vitro* modified DCs. In parallel, the same configuration of CSCs markers positively influenced the activation status of DCs since the positive correlations were found between CSCs lacking CD29 marker and CD80^+^CD83^+^ DCs proportion. Moreover, the CD133 marker was found to exert the most significant impact on the CD80^+^CD83^+^ DCs number (Table 2).

### The proliferative effect of DCs on effector T cells in a co-culture system

Because the DC-based therapy is supposed to provoke and increase the proliferation and differentiation of cancer-specific effector lymphocytes (overcoming the cancer-associated hypo-sensitivity), we decided to conduct the co-culture of DCs with autologous effector cells for 24 hours following the stimulation of DCs with lysates or TCM. Next, we analyzed the changes in the MFI of T regulatory-like CD4^+^CD25^+^ cells and CD3^+^CD56^+^ NKT cells during cytometric analysis of all effector cells used for this test.

**Figure 7:**
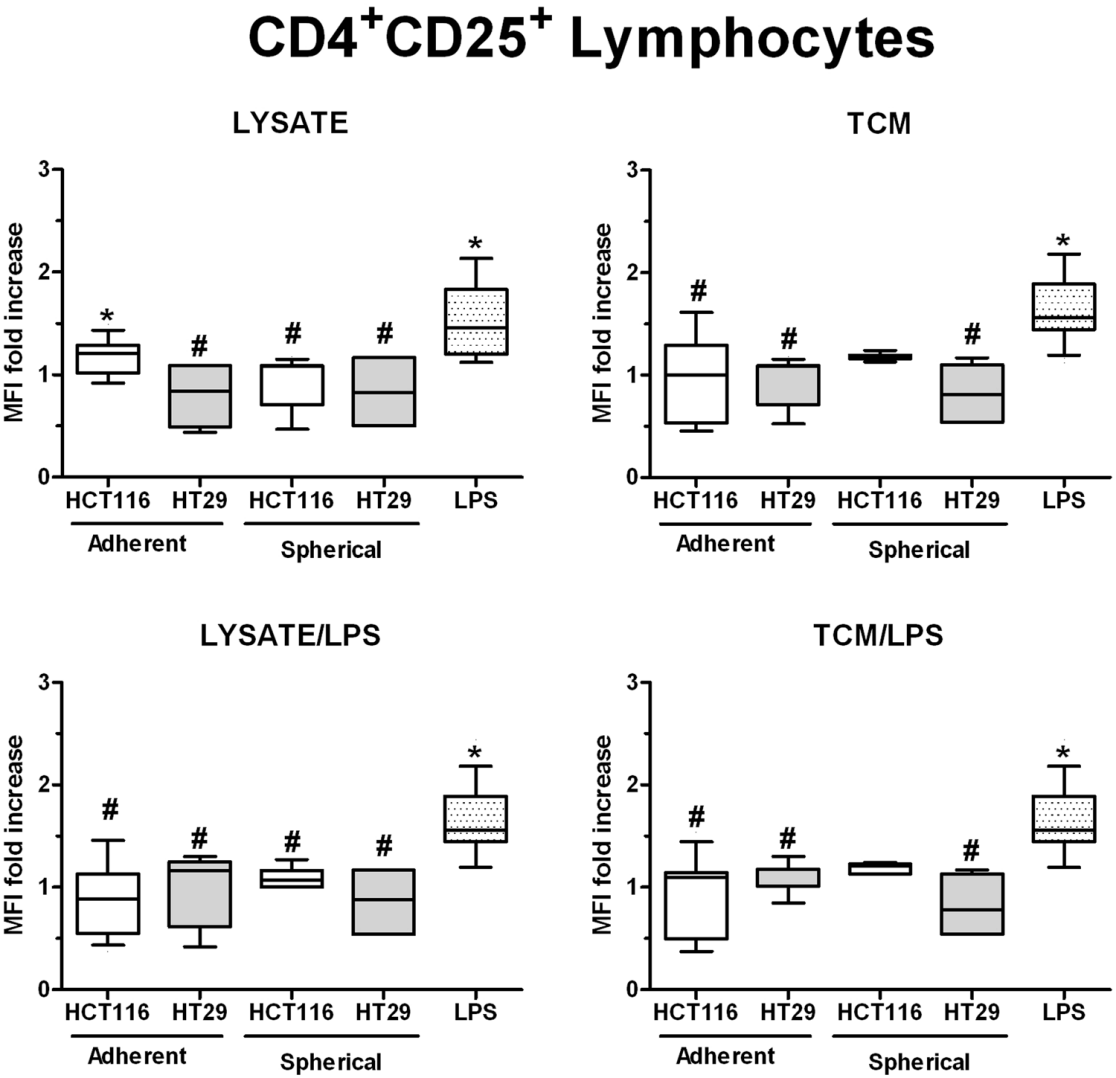
The differences in the proportion of T regulatory-like CD4^+^CD25^+^ cells after 24 h co-culture with stimulated DCs. Lymphocytes were measured amongst the effector cells acquired during the isolation of monocytic precursors for DCs expansion. Lysates and TCM used in these analyses were collected from cultures of HCT116 and HT29 cell lines expanded in adherent or spherical forms (allogenic). Data presented as MFI fold change over the level obtained after spontaneous proliferation of the control CD4^+^CD25^+^ T cells. The control value was set at the level of 1. Bars and whiskers represent mean ± SEM (*p < *0*.*05* vs iDCs, ^#^*p* < *0*.*05* vs LPS; ANOVA Kruskal – Wallis test, n = 15).

We found that DCs stimulated with all of our stimulatory agents maintained the number of T regulatory-like CD4^+^CD25^+^ lymphocytes on the same level as iDCs after co-culture. At the same time these values were lower than MFI measured for LPS-stimulated DCs (Fig. 7).

**Figure 8:**
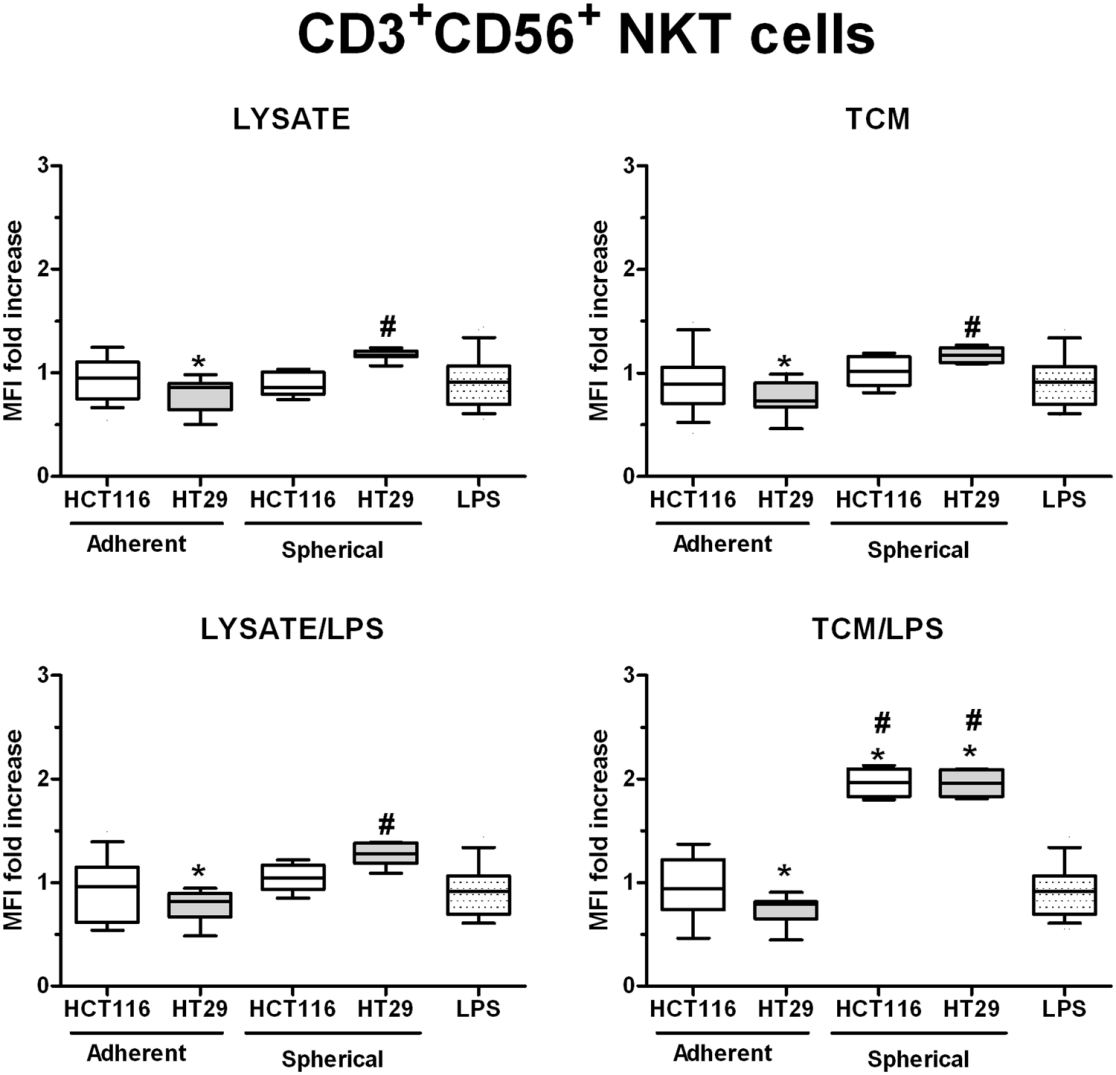
The differences in the proportion of CD3^+^CD56^+^ NKT cells after 24 h co-culture with stimulated DCs. Lymphocytes were measured amongst the effector cells acquired during the isolation of monocytic precursors for DCs expansion. Lysates and TCM used in these analyses were collected from cultures of HCT116 and HT29 CRC lines expanded in adherent or spherical forms (allogenic). Data presented as MFI fold change over the level obtained after spontaneous proliferation of the control CD3^+^CD56^+^ T cells. The control value was set at the level of 1. Bars and whiskers represent mean ± SEM (*p < *0*.*05* vs iDCs, ^#^*p* < *0*.*05* vs LPS; ANOVA Kruskal – Wallis test, n = 15).

When we analyzed the influence of stimulated DCs on the proportion of CD3^+^CD56^+^ NKT cells, we found that DCs stimulated with adherent HCT116- and HT29-derived agents could reduce the cytotoxic cells proportion in comparison to iDCs, however, only stimulation with HT-29-derived lysates and TCM exerted statistically significant effects. At the same time, the lysates and TCM originated from spherical cultures of the studied cell lines increased the number of cytotoxic cells number in comparison to iDCs and/or LPS-stimulated DCs. The most prominent elevation of CD3^+^CD56^+^ NKT cells MFI was recognized for TNM/LPS stimulation (Fig. 8). The lysates or TCM from spherical HT29 cells were the most potent to induce abilities of DCs to increase the number of NKT cells in all our stimulatory options. The MFI fold change after DCs stimulation with HT29-derived factors appeared to be even higher than values for LPS-stimulated DCs.

**Figure 9:**
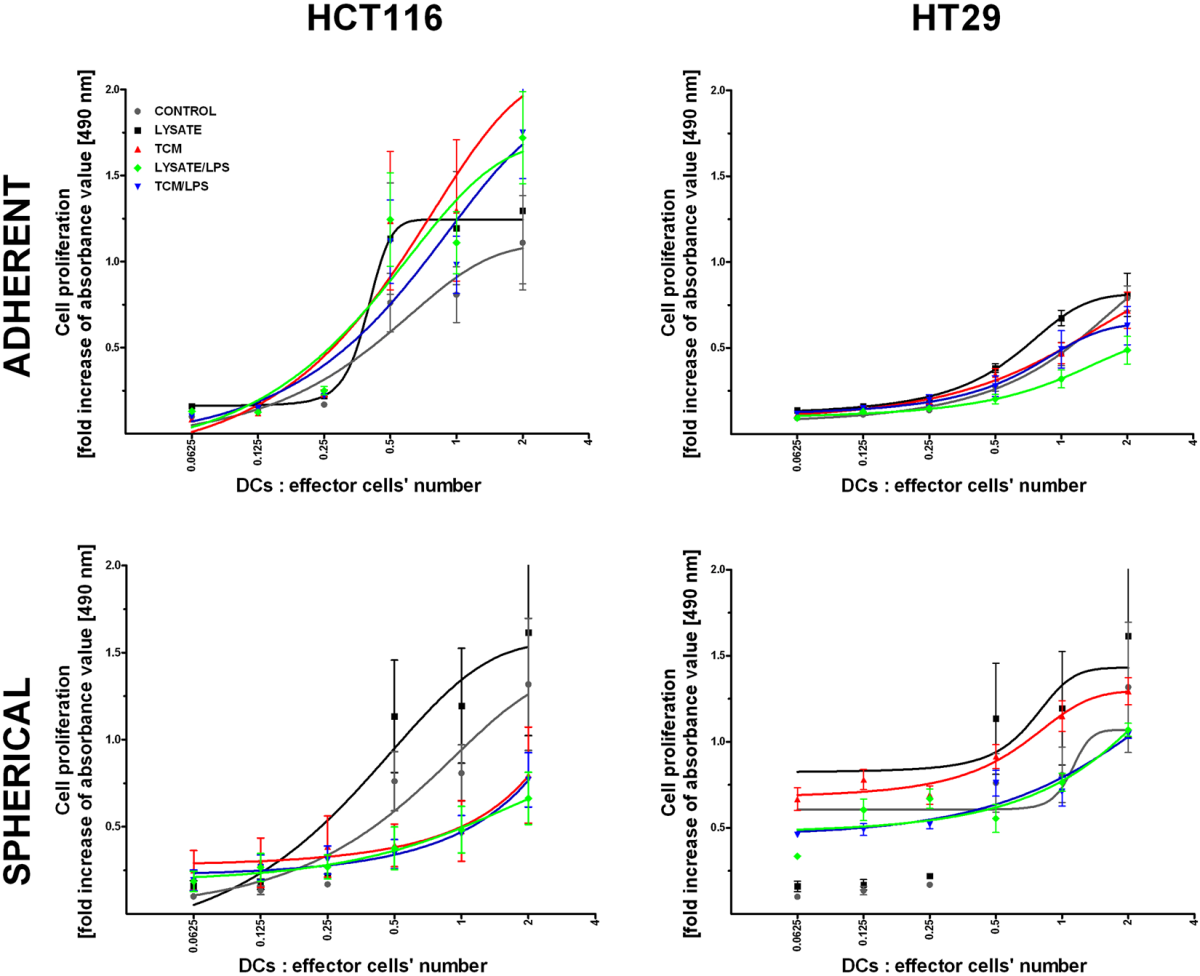
Proliferation of healthy donor-derived lymphocytes during 24 h co-cultured with stimulated DCs. Lysates and TCM used in these analyses were collected from cultures of HCT116 and HT29 CRC lines expanded in adherent or spherical forms (allogenic). Data presented as fold change over the level obtained after spontaneous proliferation of the control lymphocytes. Bars and whiskers represent mean ± SEM (*p < *0*.*05* vs iDCs, ANOVA Kruskal – Wallis test, n = 15).

The co-culture of stimulated DCs from CRC patients or healthy volunteers with their specific effector cells (CD14^−^ cells or nonadherent cells, respectively, as described in Material and Methods paragraph) was followed by the colorimetric proliferative assay to find out if DCs could change the proliferative potential of the effector cells (Fig. 9). Generally, only cancer cells lysate-stimulated DCs could increase proliferative status of effector cells after co-culture with DCs. The same observation was made for DCs treated with lysates of all our CRC cell lines (in adherent and spherical forms). Lysate-stimulated DCs induced proliferation of lymphocytes whereas the effect of other stimulators depended on origin (HCT116 versus HT29) or culture form (adherent versus spherical). The most intense proliferation was induced by DCs stimulated with adherent HCT116 cells. At the same time, HT29 cells presented opposite effects since adherent cell-derived TCM, TCM/LPS and lysate/LPS lowered the abilities of DCs to induce lymphocytes proliferation below iDCs (CONTROL) level. Interestingly, DCs treated with lysates and TCM collected from spherical cultures of both CRC lines used simultaneously with LPS decreased the number of effector cell divisions. Additionally, the CRC CSC-derived lysates and TCM in the combination with LPS exhibited similar effects on DCs functions since we found lower number of effectors after stimulation (data not showed).

### The endocytotic activity of *in vitro* modified DCs

Differentiation of monocytes into immature DCs is accompanied by the increase of their capacity to take up some foreign antigens. However, the maturation of iDCs to DCs decreases this particular activity since in mature DCs in which the function of antigen-presenting cells (APCs) prevails^26^. The endocytotic capacity was measured by flow cytometry with the use of dextran particles coupled with FITC.

**Figure 10:**
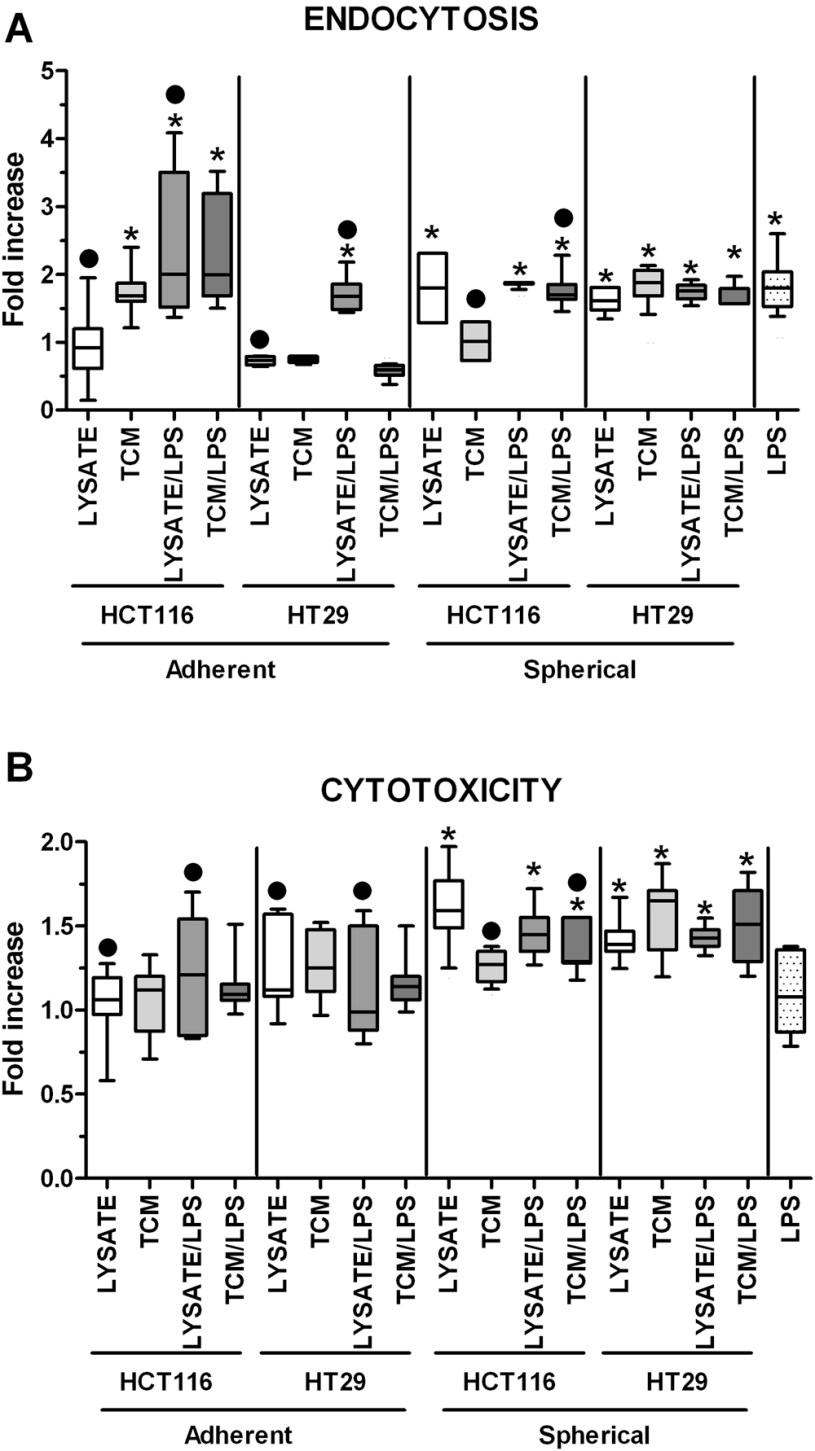
**(A)** The endocytosis effectiveness of dextran-FITC particles by stimulated DCs. Lysates and TCM used in these analyses were collected from cultures of HCT116 and HT29 CRC lines expanded in adherent or spherical forms (allogenic). Data are presented as fold increase of the proportions of dextran-FITC^+^ cells in comparison to iDCs after incubation with dextran-FITC particles. The control value was set at the level of 1. Bars and whiskers represent mean ± SEM (*p < *0*.*05* vs iDCs, ^•^*p* < *0*.*05* lysate vs lysate/LPS or TCM vs TCM/LPS, ANOVA Kruskal – Wallis test, n = 15). **(B)** The viability of HCT116 cells after 24 h co-culture with stimulated DCs. The fold increase of CD133^+^7AAD^+^cells proportions was determined by flow cytometry by comparison of stimulated DCs with iDCs. The control value was set at the level of 1. Bars and whiskers represent mean ± SEM (*p < *0*.*05* vs iDCs, ^•^*p* < *0*.*05* lysate vs lysate/LPS or TCM vs TCM/LPS, ANOVA Kruskal – Wallis test, n = 15).

The most effective endocytosis was observed for DCs stimulated with adherent HCT116-derived agents, significantly higher than in iDCs and LPS-stimulated DCs. The cancer cells derived from adherent and spherical cultures triggered different effects on the analyzed phenomenon and these differences were cell line-specific (Fig. 10A). HCT116 cells in their adherent form and HT29 cells in their spherical form presented more efficient potential to increase endocytosis by stimulated DCs in comparison to cells cultured in the other form. Surprisingly, adherent HT29 cells were the only ones in our experimental setting enabled to lower the endocytotic activity of DCs (below iDCs level) and this effect was the most prominent for TCM/LPS-stimulated DCs (Fig. 10A).

When we analyzed the correlations between phenotype of CRC lines-derived CSCs and endocytotic activity of stimulated DCs, we found no relationships between those parameters when adherent lines were taken for DC stimulation. On the contrary, when we used spherical CRC cells to induce DCs maturation, the numerous correlations were identified. Surprisingly, lysates and TCM exerted opposite effects. Lysate/LPS seemed to enhance the endocytotic activity of DCs regardless of CSCs phenotype whereas DCs treated with TCM/LPS demonstrated diverse effectiveness of particulate material uptake. The proportion of CD133^−^CD44^+^CD29^−^ cells within whole cancer cells population correlated negatively with the endocytotic activity of DCs stimulated with TCM (R = −0.65, *p* < *0*.*05*). In parallel, the CD133^−^CD44^−^CD29^+^ cells exerted the opposite effects since the correlation rank was positive (R = 0.66, *p* < *0*.*05*). These data suggest that the presence of CD44 protein on a surface of CSCs seemed to lower the endocytotic activity of DCs when used for DC stimulation. For the population of CD133^+^ CSCs we could observe similar tendencies.

Additionally, our results demonstrated that the higher the proportion of CD11c^+^HLA-DR^+^ and CD80^+^CD83^+^ DCs after specific stimulation with TCM, the higher endocytotic activity of these DCs (R = 0.49, *p* < *0*.*05*) found. Nonetheless, lysate/LPS appeared to decrease the antigen uptake simultaneously with increased proportion of activated DCs (R = −0.47, *p* < *0*.*05*).

### The viability of cancer cells after co-culture with *in vitro* modified DCs

In order to evaluate the direct cytotoxic potential of DCs obtained during our procedures, we conducted co-cultures of healthy donors blood monocyte-derived DCs with HCT116 and HT29 cells in both adherent and spherical forms.

The proportion of dead CD133^+^7AAD^+^ cancer cells after incubation with DCs treated with adherent cancer cell-derived agents was similar to the effect triggered by iDCs. The exceptions were DCs stimulated with HT29-derived TCM and HCT116-derived lysate/LPS (Fig. 10B). Spherical counterparts of both CRC lines, but especially HT29, presented significant effects due to prominent elevation of dying cancer cells proportion measured after co-culture, what suggested that DCs were able to eliminate cancer cells more effectively.

Surprisingly, after the analysis of the correlations between DCs phenotype and their cytotoxic properties, we could notice the positive relationship only within DC populations stimulated with spherical culture-derived agents, especially with TCM/LPS (R = 0.50, *p* < *0*.*05*). Similarly, the spherical culture-derived TCM in the cooperation with LPS exerted positive effect on the abilities of DCs to increase the proportion of NKT cells (R = 0.58, *p* < *0*.*05*). Surprisingly, the adherent CRC cell lines seemed not to influence DCs cytotoxic potential.

### The analysis of the soluble proteins concentration in supernatants from cultures of stimulated DCs

We also assessed the ability of stimulated DCs to secrete proteins which are crucial for DCs different specialization profiles: IL-10 and IFNγ – for suppressive DCs, IL12p70 – for pro-inflammatory DCs, granzymes – for cytotoxic DCs and sFasL – for DCs able to induce apoptosis of FasR^+^ target cells.

Generally, DCs incubated with two agents simultaneously (lysate/LPS and TCM/LPS) presented the highest secretory efficiency. Moreover, DCs in our study were the most potent to secrete IL-12p70 from all measured soluble proteins (Fig. 14). The highest potential to induce secretory activity of DCs was showed for adherent and spherical HT29-derived factors.

**Figure 11:**
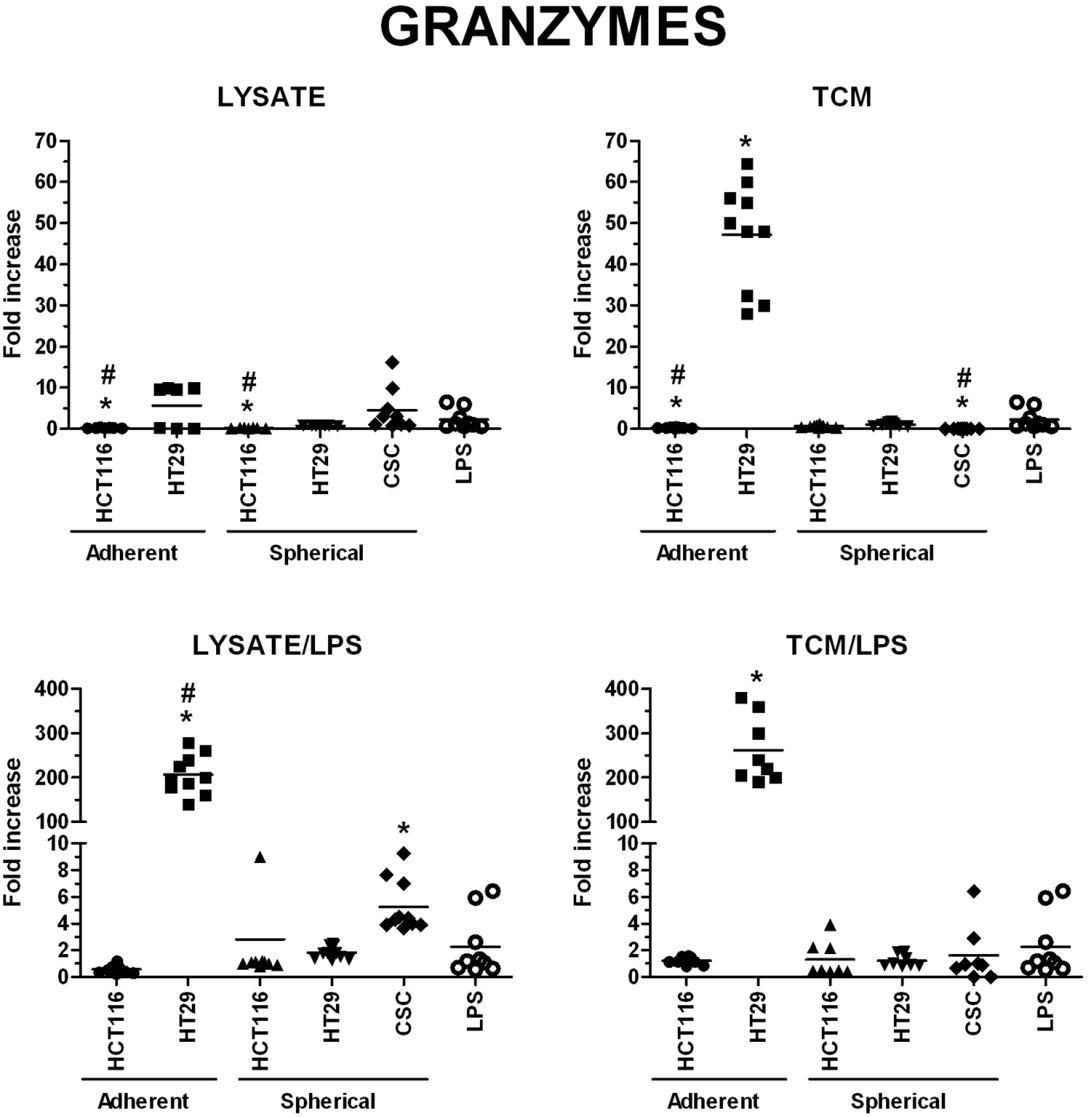
The granzymes concentration [pg/ml] in supernatants collected during the culture of stimulated DCs (n = 8–10). Data presented as fold change over the level obtained from iDCs culture. Lysates and TCM used in these analyses were collected from cultures of HCT116 and HT29 CRC lines expanded in adherent or spherical forms (allogenic), and CRC patients-derived CSCs (autologous). Bars represent median value (*p < *0*.*05* vs iDCs, ^#^*p* < *0*.*05* vs LPS; ANOVA Kruskal – Wallis test, n = 15).

**Figure 12:**
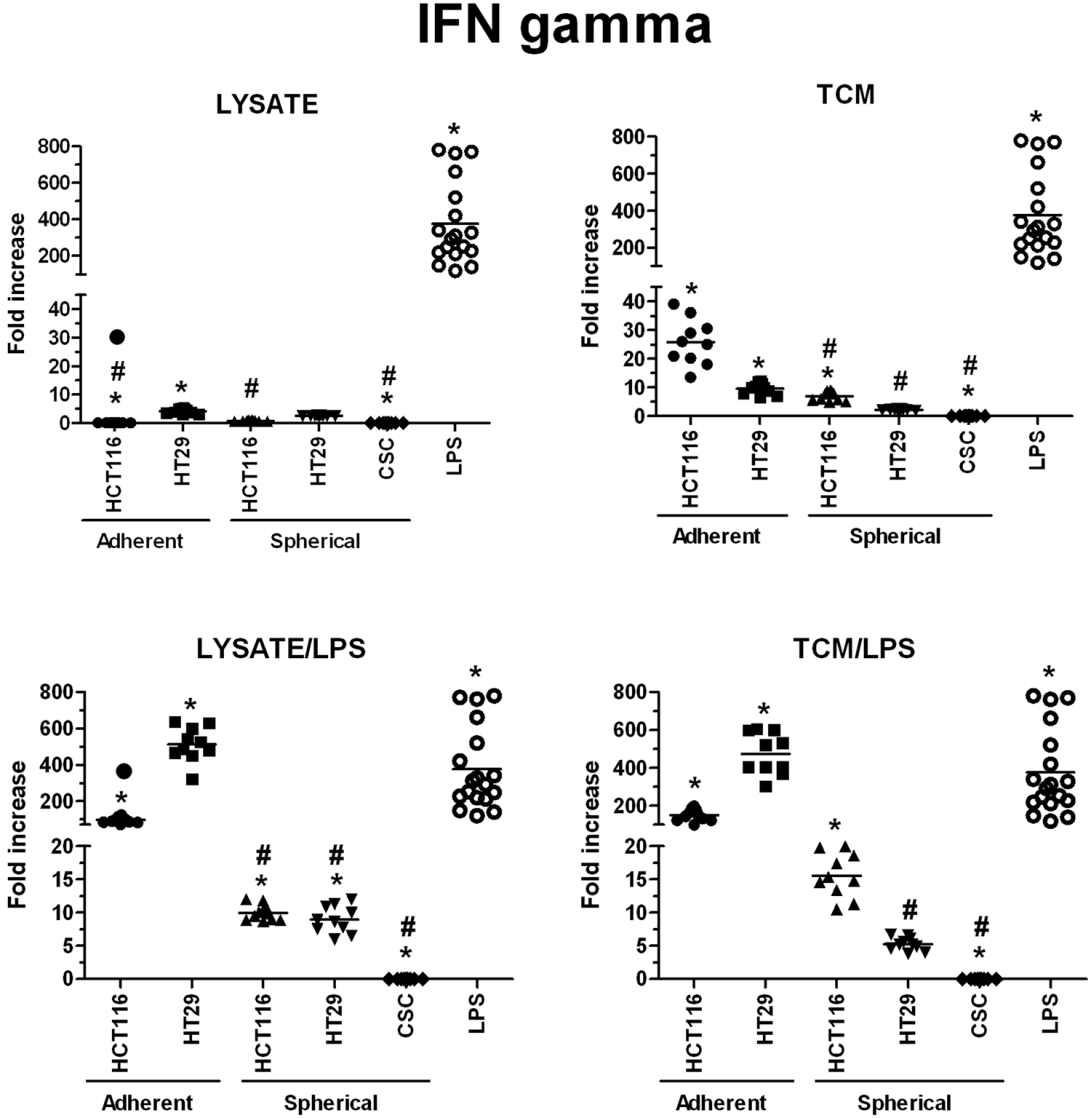
The IFNγ concentration [pg/ml] in supernatants collected during the culture of stimulated DCs (n = 8–10). Data presented as fold change over the level obtained from iDCs culture. Lysates and TCM used in these analyses were collected from cultures of HCT116 and HT29 CRC lines expanded in adherent or spherical forms (allogenic), and CRC patients-derived CSCs (autologous). Bars represent median value (*p < *0*.*05* vs iDCs, ^•^*p* < *0*.*05* lysate vs lysate/LPS or TCM vs TCM/LPS, ^#^*p* < *0*.*05* vs LPS; ANOVA Kruskal – Wallis test, n = 15).

**Figure 13:**
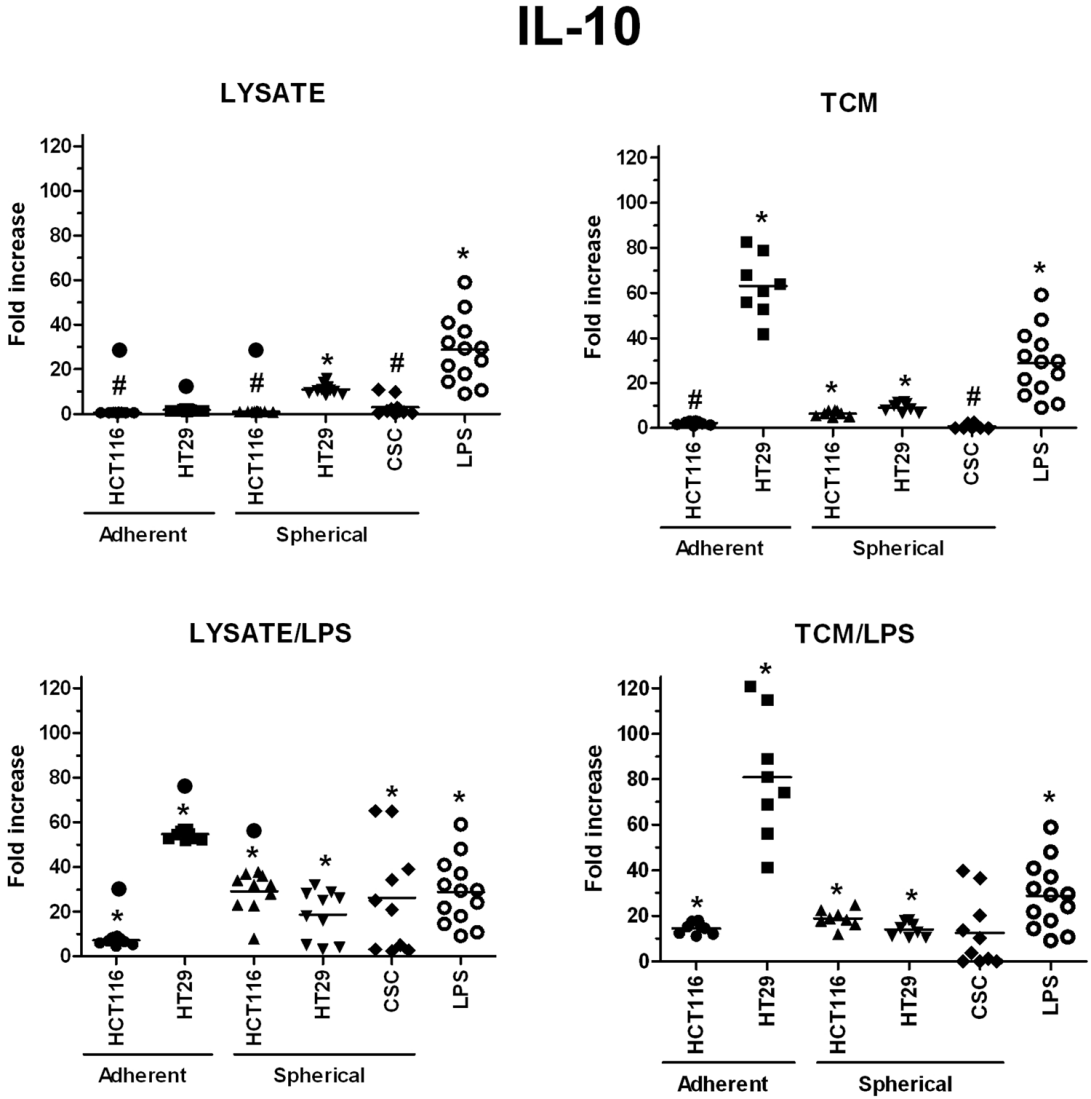
The IL-10 concentration [pg/ml] in supernatants collected during the culture of stimulated DCs (n = 8–10). Data presented as fold change over the level obtained from iDCs culture. Lysates and TCM used in these analyses were collected from cultures of HCT116 and HT29 CRC lines expanded in adherent or spherical forms (allogenic), and CRC patients-derived CSCs (autologous). Bars represent median value (*p < *0*.*05* vs iDCs, ^•^*p* < *0*.*05* lysate vs lysate/LPS or TCM vs TCM/LPS, ^#^*p* < *0*.*05* vs LPS; ANOVA Kruskal – Wallis test, n = 15).

**Figure 14:**
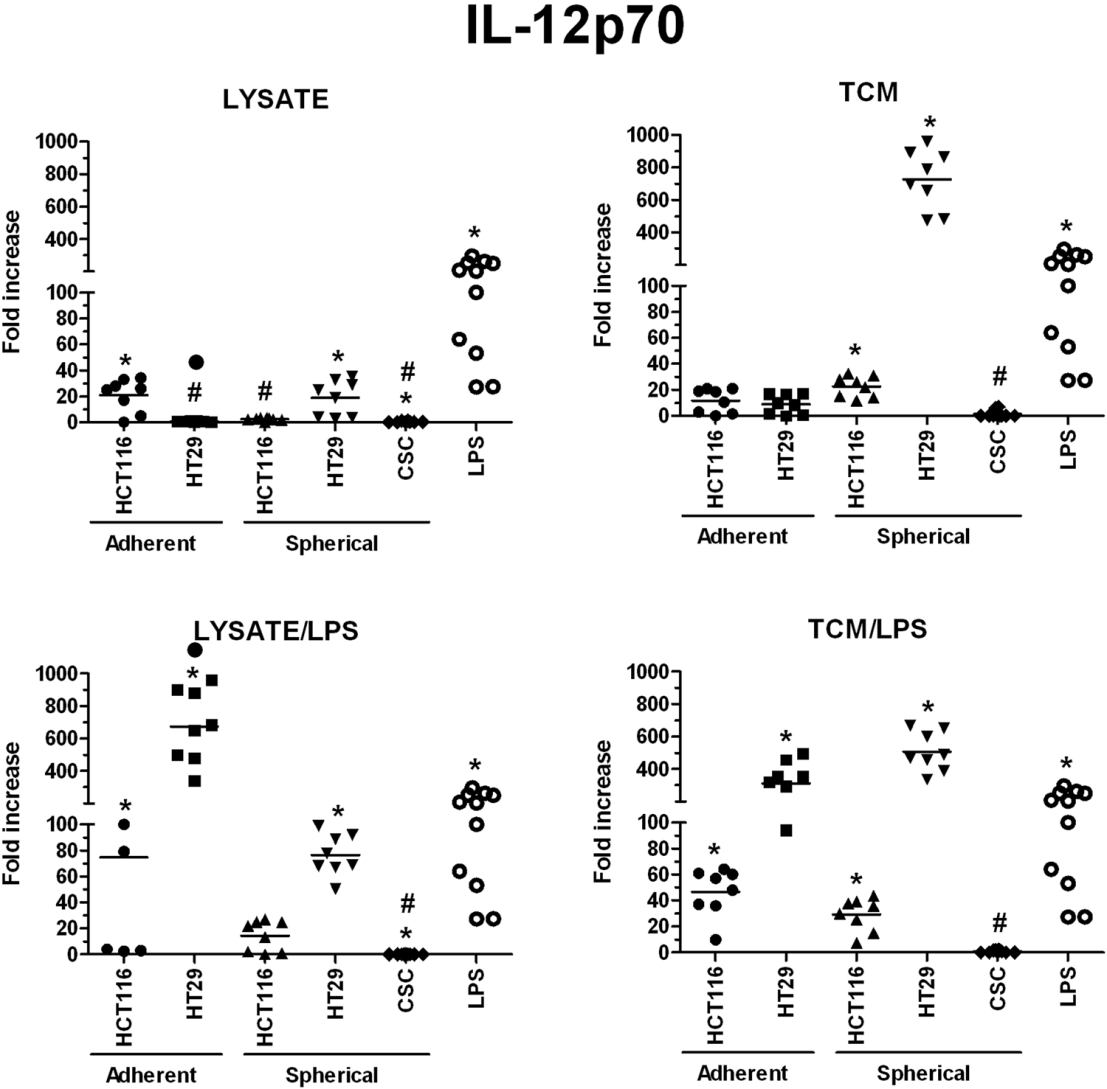
The IL-12p70 concentration [pg/ml] in supernatants collected during the culture of stimulated DCs (n = 8–10). Data presented as fold change over the level obtained from iDCs culture. Lysates and TCM used in these analyses were collected from cultures of HCT116 and HT29 CRC lines expanded in adherent or spherical forms (allogenic), and CRC patients-derived CSCs (autologous). Bars represent median value (*p < *0*.*05* vs iDCs, ^•^*p* < *0*.*05* lysate vs lysate/LPS or TCM vs TCM/LPS, ^#^*p* < *0*.*05* vs LPS; ANOVA Kruskal – Wallis test, n = 15).

**Figure 15:**
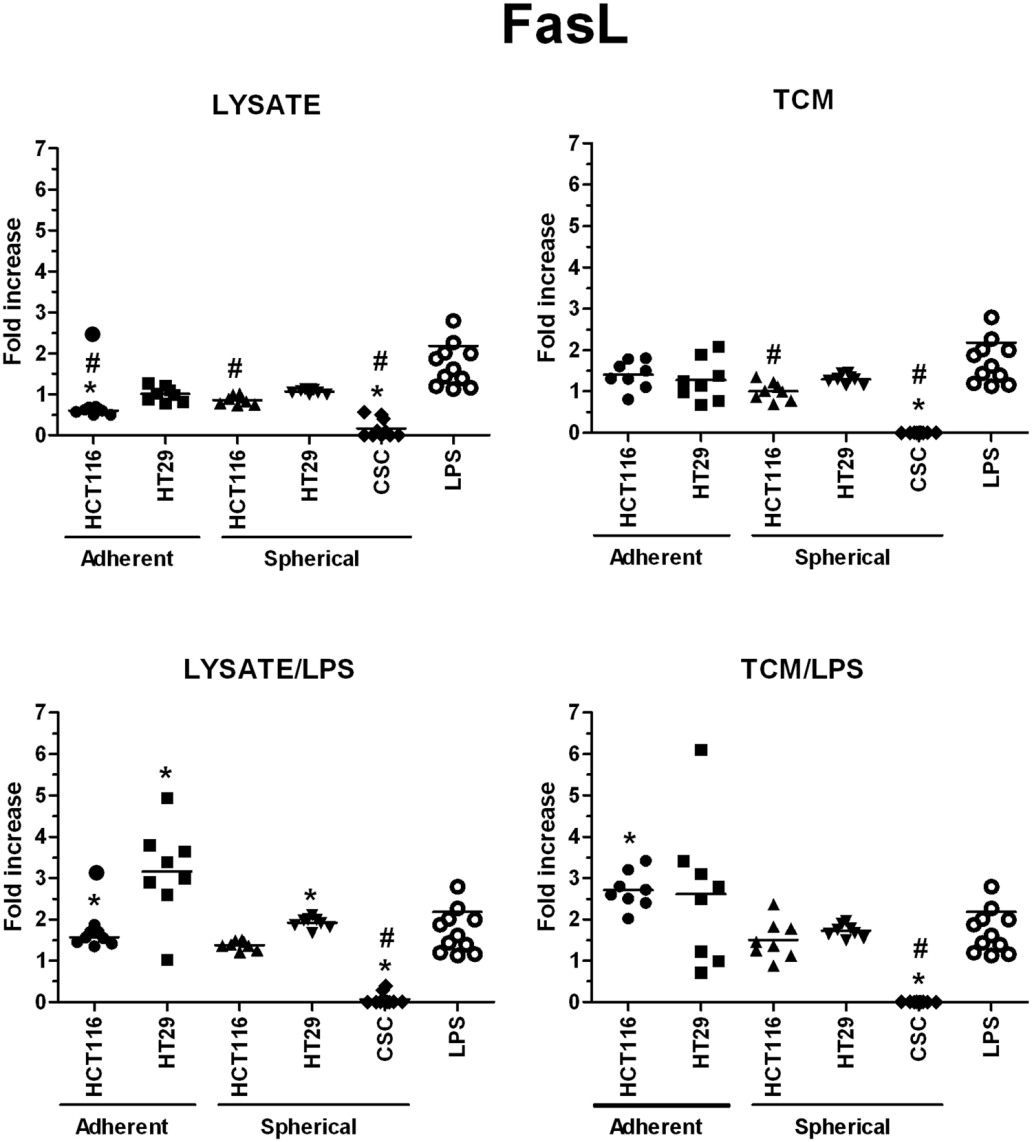
The sFasL concentration [pg/ml] in supernatants collected during the culture of stimulated DCs (n = 8–10). Data presented as fold change over the level obtained from iDCs culture. Lysates and TCM used in these analyses were collected from cultures of HCT116 and HT29 CRC lines expanded in adherent or spherical forms (allogenic), and CRC patients-derived CSCs (autologous). Bars represent median value (*p < *0*.*05* vs iDCs, ^•^*p* < *0*.*05* lysate vs lysate/LPS or TCM vs TCM/LPS, ^#^*p* < *0*.*05* vs LPS; ANOVA Kruskal – Wallis test, n = 15).

The highest concentrations of granzymes were found in supernatants from the cultures of HT29-dervived lysates and TCM-stimulated DCs, especially when lysates and TCM were administrated with LPS, the values were 230 times higher than these obtained for iDCs (Fig. 11). When we compared the granzymes concentrations measured in cultures of DCs stimulated with spherical CRC cells-derived factors, we surprisingly found that CRC patient-derived lysates, lysate/LPS and TCM/LPS caused more active secretion of granzymes by DCs than spherical HCT116 and HT29-derived compounds (Fig. 11). Next, the secretory activity of DCs treated with CRC patients-derived agents was examined and we found that IFNγ (Fig. 12), IL-12p70 (Fig. 14) and sFasL (Fig. 15) were lower in comparison to iDCs, however these DCs were able to significantly increase exocytosis of IL-10 after incubation with lysate/LPS or TCM/LPS (Fig. 13).

The lowest values of secreted proteins were obtained for soluble form of FasL since in many cases we even found its concentration to decrease below the level acquired by iDCs (Fig. 15). Only adherent CRC lines could exert positive effect in regard to the sFasL secretion during culture, whereas spherical cells of both CRC lines and patients solid tumors lowered exocytosis of this potent pro-apoptotic agent.

IFNγ was the most effectively secreted by DCs stimulated with lysate/LPS or TCM/LPS originated from both adherent CRC cell lines (Fig. 12) whereas the spherical cancer cell-derived factors presented much weaker impact on IFNγ secretion. IL-12p70 was found at the highest concentration in supernatants collected from DCs stimulated with the use of HT29-derived lysate/LPS or TCM/LPS and TCM or TCM/LPS – from adherent and spherical forms of CRC cell lines, respectively. These levels were many times higher than levels induced by factors from different types of cultures (Fig. 14).

We found the highest concentrations of IL-10, IL-12p70, granzymes and IFNγ in cultures of DCs stimulated with adherent HT29-derived lysate/LPS and TCM/LPS. The most dominating seemed to be the secretion of proinflammatory IL-12p70 (up to 667-fold increased concentration than iDCs), but also granzymes (up to 230x) were found outstanding.

## Discussion

In order to evaluate the DCs suitability for anti-cancer regiments, the first requirement is to get a system in which they could be modified and propagated. Since it was found in cancer patients that DCs mature defectively or they could even be kidnapped to support the cancer progression^67–69^, it became crucial to find an alternative source of fully effective DCs for clinical use^29,70–72^. Another problem is a limited access to monocytic precursors from individuals with advanced cancer. The characterization of the optimal environment supporting development of effective DCs subpopulations requires relatively high number of these cells. We found out that leukocytes from healthy volunteers can provide monocytic precursors which^64^ can be in relatively simply way differentiated toward DCs with the use of GM-CSF and IL-4. The isolation and *in vitro* differentiation of circulating monocytic precursors enabled us to establish the appropriate methodology for specific evaluation of anti-cancer properties of *in vitro* modified DCs. The clinical application of DC-based procedures for the treatment of cancers with low immunogenicity including colorectal^73^, prostate and renal^74^ cancers and hematological neoplasms^75^ has been previously reported. *In vitro* modified DCs applied as monotherapy for cancer patients were found to be safe and rarely induced toxic side effects in contrast to treatments that used cytokines and other biologically active molecules^64,76,77^.

Since we conducted most of the laboratory tests simultaneously on CSCs isolated from cancer tissue of CRC patients and on two commercially available CRC cell lines (HCT116 and HT29), it was possible to compare corresponding panels of DCs activities. Moreover, we expanded CRC lines in two distinct forms: adherent (recommended by supplier and historically first) and spherical, which is thought to resemble *in vivo* environment for CSCs survival^37^. Such diversification of the cellular forms allowed us to directly compare the usefulness of both forms of CRC cell lines for the *in vitro* investigations. According to our best knowledge no other study has addressed the evaluation of specific DCs activities in such an ample repertoire of *in vitro* experiments.

As we reported earlier^36^, the adherent forms of the studied HCT116 and HT29 CRC cell lines are characterized by 100%-enrichment in CD133^+^CD44^+^CD29^+^ CSCs, however, when the culture conditions were modified into sphere-forming environment, a decrease of CD133^+^ cells number by 39% and 59% in HCT116 and HT29 cell cultures, respectively, was found. CRC patients-derived spherical cultures appeared to contain varying proportions of CD133^+^ cells (ranging from 2 to 96%). Importantly, that seemed to be related to the cells survival time in culture since we found significant correlation between the number of passages conducted on particular population and the TNM status of CRC patient who was a donor of these cells.

Primarily, our results showed superiority of the spherical culture model over the adherent one since spherical HCT116 and HT29 cells presented similar impact on DCs properties as the cancer cells collected from tumors of CRC patients. Since we could perform only a limited number of *in vitro* experiments on CRC patients-derived DCs, we used monocyte-derived DCs of healthy volunteers that were stimulated with TCM or lysates of HCT116 and HT29 CRC cell lines. The diverse results observed for DCs stimulated with CRC lines-derived lysates and TCM may be associated with different origin of these lines and epigenetic and genetic differences between them^78^. HCT116 cells represent non-differentiated and highly aggressive cell line that corresponds to the TNM III stage and HT29 line is known as more differentiated and less invasive (TNM II)^78^. The interpretation of our findings in respect of line dependency (and their origin dependency) may explain many differences between results obtained on both CRC cell lines. The obtained results were generally consistent with those conducted with DCs stimulated with agents derived from CSCs of CRC patients. For instance, stimulatory factors for DCs derived from spherical cultures of HT29 cells allowed us to obtain the higher proportions of CD11c^+^HLA-DR^+^ and slightly higher (or equal) proportions of FasL^+^ dendritic cells, less CD4^+^CD25^+^ and more CD3^+^CD56^+^ lymphocytes after co-culture with DCs, more pronounced proliferation of lymphocytes, slightly enhanced endocytosis and cytotoxicity, lower IFNγ and IL-10, higher IL-12p70 and sFasL concentrations in comparison to DCs stimulated with spherical HCT116-derived lysates and TCM. This summarized comparison suggests that the progression status of cancer significantly influenced the DCs features, probably due to more developed evasion mechanism typical for less differentiated and more aggressive cancers (such as HCT116 cell line). The CD80^+^CD83^+^ DCs number appeared to depend on stimulatory agent used during procedure. The lysates or TCM when used alone let us gain lower number of activated DCs when HT29 cells were a source of stimulatory factors in comparison to HCT116-derived agents, but the adding of LPS inverted these relationships. Moreover, we found that adherent cultures of CRC cell lines are not an appropriate model for analyzing any *in vitro* modifications and any therapeutic options for future anti-cancer protocols. Since the adherent model of cancer cells expansion is unnatural (homogenous monolayer is not a naturally occurring histological form of cancerous tissue) it should be avoided in *in vitro* cancer studies if only the cultures of cancer cells in spherical form can be obtained and processed.

The analysis of the phenotypes of CSCs and autologous DCs demonstrated that the detailed configuration of CSC-like markers may have a crucial impact on functions of stimulated DCs. The increased number of CD29^+^ and CD44^+^ CSCs presented the opposite impact on the potential of *in vitro* modified DCs since the correlations (number of CSCs vs number of DCs) presented opposite correlation ranks. The presence of CD133^+^ cells seemed to suppress DCs activities. The higher CD133^+^ CSCs proportion in patients tumor samples the lower number of activated DCs was detected after stimulation. The concomitant experiments on CRC cell lines supported this conclusion since their CD133^+^ cells analogically suppressed the features of DCs after stimulation with lysates or TCM. Moreover, the lower concentrations of granzymes and IL-12p70 in cultures of DCs derived from CRC patients with TNM III-IV stage in comparison to patients with less advanced disease (TNM I-II) deserves a special attention. Thus, our observations suggest that the progression status of colorectal cancer may play a key role for the effectiveness of *in vitro* modified DCs. Our observations may be associated with the tumor evasion phenomenon reported to complicate therapy of many different types of malignancies, including CRC^79^.

The activation and maturation of resting (immature) DCs is supposed to be associated with the series of complicated, antigen-acquisition-dependent, tightly controlled functional and phenotypic changes. DCs are aimed to acquire all essential tools for migration to lymphoid tissue and optimal activation of target-specific immune response^26^. For *ex vivo* modification strategies, the most serious pitfall is an improper DCs maturation which fails to induce antigen-specific responses and may in fact provide the immunoregulatory signals to DCs that could induce an immunosuppressive response. This limiting factor contributes to the unsatisfactory success rate of the DC vaccines^69,80–83^. This may be caused by many reasons but the salient one seems to be the insufficient knowledge about regulatory mechanisms of DC maturation and mechanisms of cancer immune evasion which may completely invert the status of injected DCs^67^. Although, our results presented diverse stances in relation to that issue, we suggest that the activation pathway of DCs triggered in our culture condition could be beneficial for the purpose of cancer immunotherapy. The stimulatory agents which were used for DC modification generally did not elevate the proportion of T regulatory-like CD4^+^CD25^+^ cells. The only exceptions were DCs stimulated with adherent HCT116-derived lysates and TCM. Additionally, the analysis of IL-10 secretion by DCs showed its low concentration in supernatants from DCs cultures in all experimental options. Unfortunately, at the same time the colorimetric assay showed the inhibition of lymphocytes proliferation during their co-culture with DCs, however, DCs stimulated by cancer cells lysates increased the number of lymphocytes in all experimental variations. The observed decreased division rates of effector lymphocytes evidenced the development of immune evasion mechanism mediated by cancer cells but DCs treated with TAA-rich lysates could avoid that according to our results and other research groups^84,85^. However, the CRC patients-derived CSCs presented the significant negative impact on DCs abilities to induce proliferation of lymphocytes during co-culture. That effect was the most prominent after DCs were stimulated with lysate/LPS or TCM/LPS. Our observation seems to confirm that CSCs are responsible for cancer evasion phenomenon since the decrease of lymphocytes division rate is one of the cancer evasion elements. The differences observed in this part of our study may be associated with the origin of lymphocytes taken for co-cultures since DCs and lymphocytes used for experiments with CRC cell lines were obtained from healthy volunteers. Lymphocytes separated from the blood of cancer patients probably were encumbered by cancer-related immunosuppresion what could have markedly implicated our final results.

Since it was earlier showed that multiple tumor-associated antigens specifically expressed by cancer cells are very efficient and produce effective anti-cancerous response *in vivo* or *in vitro*^86^, we decided to generate *in vitro* CSC-antigens-specific DCs by using either tumor conditioning medium or lysates of CSCs. Both, lysates and TCM, are known to be rich sources of a wide variety of cancer antigens necessary for DCs activation. The analysis of DC phenotype after their incubation with TCM or lysates from CSCs revealed that the proportions of CD11c^+^HLA-DR^+^ and CD80^+^CD83^+^ activated effector DCs depended on the stimulatory agent used. The basic stimulatory effect with TAA-rich lysates or TCM provided some kind of leading signal for the whole maturation but LPS seemed to intensify these effects. The usage of LPS in our experimental procedures seemed to be appropriate for colon-derived cells bacause the presence of LPS-rich bacteria is the common physiological phenomenon for this particular niche. For that reason our results suggest that LPS should be introduced into analyses of anticancer activities of DCs in *in vitro* studies.

The cancer cell-derived lysates were confirmed to be a strong immune stimulus for therapeutic use of *ex vivo* generated DCs^83^. Other researchers applied γ-irradiated dying or staurosporin-treated HT29 cells which after being engulfed during phagocytosis were more potent in DC activation than cell lysates^87^. In our study lysates alone induced weaker effects on DCs functions than lysates with LPS because lysate/LPS provided significantly enhanced stimulation especially in regard to DC phenotype, their ability to kill cancer cells during co-culturing and to secrete cytokines. Our results showed that apart from applying lysates for DC stimulation, the additional support is necessary to achieve the more prominent effect of treatment.

O’Toole’s^88^ study conducted on samples of CRC patients tumors, showed that TCM added to DC culture did not affect cytokine secretion or DC phenotype when compared to iDCs indicating constant hypo-responsiveness of CRC patients DCs. That is partially in accordance with our study which demonstrated that TCM inhibited patients-derived DCs secretory activity. However, these DCs presented significantly higher proportion of mature CD11c^+^HLA-DR^+^ DCs in comparison to iDCs. At the same time the numbers of CD80^+^CD83^+^ DCs and FasL^+^ DCs maintained at the same level as within iDCs. Additionally, experiments conducted with the use of spherical CRC lines indicated some differences in comparison to these made with CRC patients cells. We could observe elevated proportions of matured and activated DCs, but TCM only in the combination with LPS exerted statistically significant effect. In contrast, lysates (especially in the combination with LPS) of HCT116 and HT29 cells significantly stimulated DCs activation, even to a larger extent than LPS alone.

However, in the functional tests assessing endocytotic and cytotoxic activities of DCs, the results appeared to be more heterogeneous and were CRC line-dependent. The proportion of NKT cells (CD3^+^CD56^+^) was significantly enhanced after incubation with DCs stimulated with TCM/LPS from spherical cultures of both CRC cell lines. These results were found positive in regard to the goals of our study since the increased proportion of CD3^+^CD56^+^ NKT cells was demonstrated earlier as one of the most desirable effects of DC-based therapy^2,89^. The Lindenberg group^90^ published similar observations after they stimulated skin DCs with supernatants from the 3-D culture of CRC cells from cancer patients.

Our observations indicated that spherical forms of CRC cells cultures presented potential to modify DCs activity but it depended on type of CRC cell line used for the procedure. Importantly spherical cell lines presented potential to modify DC activity which was more comparable to potential exerted by patients-derived CSCs in comparison to adherent lines. It was evidenced by the secretory activity of DCs (especially sFasL, IL-10, and granzymes) which displayed similar tendencies in cultures of DCs treated with both CRC patients-derived and spherical CRC lines-derived stimulatory factors. At the same time, the secretion of IFNγ and IL12-p70 appeared to be more intense after the stimulation with lysate/LPS and TCM/LPS derived from spherical CRC lines in comparison to stimulated DCs of CRC-patient origin. The more effective secretion of IL12-p70 after DCs stimulation with spherical HT29 cells in comparison to stimulation with HCT116 cells is consistent with the results concerning DCs of cancer patients. Individuals with less advanced CRC (TNM I – II) exhibited higher secretion of this proinflammatory protein in comparison to patients with TNM III-IV stage whereas HT29 cells are referred to as TNM II while HCT116 – TNM III.

Moreover, we identified numerous correlations between various cellular parameters and these relationships were significant when TCM and lysates from spherical cultures were used for DC stimulation. We feel that the negative correlations between the number of HCT116 and HT29 spherical cancer cells with specific phenotype and DCs endocytotic activity deserve a special attention. These relationships confirmed the negative impact of cancer cells bearing the CSC-like markers on the effect of *in vitro* DC stimulation, on how effectively DCs mature and conduct endocytosis.

Because the elevated proportion of FasL^+^ DCs was not observed during our study and the concentration of soluble FasL was extremely low, we could conclude that FasL was not engaged in the cytotoxicity against FasR^+^ lymphocytes and CRC cancer cells under the conditions of our experiments. Moreover, the equal or even lower number of FasL^+^ DCs in comparison to iDCs confirmed the cancer evasion capabilities of CRC cells.

We showed that tumorsphere assay-based drug evaluation of anti-cancer agents will provide more translatable results compared to those obtained from the traditional 2-D monolayer culture system. Our observations, a first of that type, contribute to the expansion of knowledge about the use of DCs in the studies on cancer biology, with special focus on cancer stem cells. It is hoped that the obtained results can assist in efforts to successful clinical application of some novel anti-cancer DC-based therapies.

However, our results show that in addition to lysates and TCM, the additional support is necessary to achieve the desired effect of DC *in vitro* modifications. For the CRC niche the usage of LPS seems to be justified and beneficial. In view of the strong immunosuppression, which accompanies neoplastic diseases, unmodified DCs are not sufficient to effectively stimulate a cellular response. The use of *in vitro* modified DCs in cancer therapy is certainly an interesting and promising direction of research. However, the protocols of generation and stimulation of DCs with tumor antigens should be worked out before vaccination of patients is introduced into standard therapy.

## Material and Methods

### Study design

Freshly isolated colorectal cancer tissues were used to isolate and expand CSCs. CSCs were also isolated and expanded from two CRC cell lines, HT116 and HT29, that were cultured both in adherent and spherical forms. Peripheral blood of CRC patients and healthy volunteers was used to isolate and expand dendritic cells from their monocytic precursors. Autologous lymphocytes were also used for co-cultures with dendritic cells. At the end of expansion of DCs they were modified with lysates or tumor conditioned media or LPS as well as the combinations of these stimulants. Furthermore, the biological properties of DCs subjected to these procedures were determined.

### Isolation and expansion of CRC primary cell lines

All human tissues samples (cancer and peripheral blood) used in the study were donated freely and written informed consent was obtained from all patients. Ethical approval was obtained from The Independent Bioethics Commission for Research of the Medical University of Gdansk and all research was performed in accordance with relevant guidelines. The general clinicopathological characteristics of CRC patients were presented in the Table 1. Among our study population the cancer was localized in cecum (27%), ascending colon (13%), transverse colon (7%), descending colon (7%), sigmoid (33%), rectum (20%), colic flexures (10%) and 20% of patients had diagnosed CRC within two different segments of large bowel. Additionally, among them 14 had developed lymph node metastasis (TNM stage III) and 3 had distant metastasis (TNM stage IV) at the time of diagnosis.

**Table 1:** Clinicopathological characteristics of CRC patients included into a study.

TNM stage	I-II	III-IV
N	5	13
Gender	F 1	F 8
	M 4	M 5
Age (years)	74	75
Mean [min-max]	[67–80]	[63–88]
CD133^+^ [%]	9	68*
Mean [min-max]	[2–20]	[34–96]
FasL^+^ [%]	56	78*
Mean [min-max]	[53–59]	[54–96]
Proliferation rate^1^	1	1.2*
Mean [min-max]	[0.6–1.4]	[0.6–2.9]

F-females, M-men. *significantly different from the TNM I-II group, *p* < *0*.*05*, U Mann-Whitney test; ^1^fold increase of the number of CD133^+^ cells measured during passage.

**Table 2:** Correlations between the proportion of CD11c^+^HLA-DR^+^ and CD80^+^CD83^+^ DCs and DCs endocytotic activity and the phenotype of cancer cells which were the source of lysates or TCM for given analysis (*p < *0*.*05*, Spearman’s rank correlation coefficient).

	DC CD11c^+^HLA-DR^+^				DC CD80^+^CD83^+^			
	LYSATE	TCM	LYSATE /LPS	TCM /LPS	LYSATE	TCM	LYSATE /LPS	TCM /LPS
CD133^+^	−0.44	−0.08	−0.15	−0.15	−0.72^*^	−0.62^*^	−0.76^*^	−0.77^*^
CD133^+^CD44^+^CD29^+^	0.18	0.25	0.22	0.24	−0.28	−0.15	−0.60^*^	−0.59^*^
CD133^-^CD44^+^CD29^+^	0.41	0.22	0.54	0.50	0.22	0.40^*^	0.00	0.00
CD133^-^CD44^+^CD29^-^	−0.62^*^	−0.63^*^	−0.98^*^	−0.97^*^	−0.46	0.03	0.39	0.41
CD133^-^CD44^-^CD29^+^	−0.12	−0.06	−0.38	−0.38	−0.48^*^	−0.37^*^	−0.38^*^	−0.39^*^

Lysates and TCM (tumor conditioned medium) used in these analyses were collected from cultures of HCT116 and HT29 CRC lines expanded in spherical form (allogenic) and CRC patients CSCs in the form of spheroids (autologous).

Freshly resected colorectal cancer tissues from 27 CRC patients treated in the Department of General, Endocrine and Transplant Surgery, Medical University of Gdansk, Poland, were collected and immediately processed in culture. All experimental chemicals were purchased from SigmaAldrich, Poznan, Poland, except for growth factors, which were purchased from R&D Systems, Biokom, Warszawa, Poland. Tissues were washed several times in serum-free Dulbecco’s modified Eagle medium (DMEM) - F12 supplemented with antibiotic-antimycotic agents. The specimens were minced into 1–2 mm3 pieces followed by incubation in collagenase (20 ng/ml) and hyaluronidase (20 ng/ml) for 1.5 h at 37 °C. Single cell suspension was obtained by mixing every 15 minutes and by filtration through a 70 μm cell strainer.

Primary colon spheroid cultures (SC) were treated as we described previously^36^. Briefly, SCs were maintained on low adherent culture flasks and plates in serum-free stem cell medium containing DMEM-F12 supplemented with ITS Liquid Media Complement (1x), BSA (4 mg/ml), glucose (3 ml/ml), Hepes (5 mM), L-glutamine (2 mM), progesterone (20 nM), putrescine (9.6 µg/ml), Heparin (4 μg/ml), EGF (20 ng/ml), bFGF (20 ng/ml), and antibiotic-antimycotic solution (1x). For the need of current study, we used early passaged (from third to fifth passage) SCs for analysis. During each passage the medium was collected and stored in −80 °C for DC stimulation and was referred to as tumor conditioned medium (TCM).

For passage spheroid cultures were collected into the tube, centrifuged and resuspended in fresh SC medium following rigorous pippeting aimed to dissociate the spheres into cells suspension.

### Expansion of HCT116 and HT29 cell lines in adherent and spherical cultures

The HT29 and HCT116 human colon adenocarcinoma cell lines (obtained originally from the American Type Culture Collection (ATCC), Manassas, VA, USA) were used in this study. The cells were cultured routinely as a monolayer in McCoy’s medium supplemented with fetal bovine serum (10%), penicillin-streptomycin (1%) and L-Glutamine (2 mM) and incubated at 37 °C under a humidified atmosphere of 5% CO_2_. The cells were serially treated with trypsin when they achieved 80% confluence and the medium was renewed 2–3 times per week.

For the spheroid culture of HCT116 and HT29 cell lines, they were grown in SC medium characterized in previous paragraph. Cancer cultures were maintained under these conditions for 5–6 passages before being used for experiments. During each passage the TCM was collected and stored in −80 °C for DCs stimulation.

### Generation and expansion of DCs from peripheral blood monocytes of healthy donors

We used leukocyte-platelet coats (*n* = 36) obtained from volunteers recruited during the routine medical consultation in the Regional Blood Bank in Gdansk, Poland, and only the healthy individuals were included into this study. Peripheral blood mononuclear cells were separated by Histopaque^®^−1077 gradient centrifugation at 1200 *g*, 30 min, RT. After isolation and erythrocytes lyses, cells were washed and prepared to further isolation steps. To separate monocytes, PBMCs were cultured for 24 h on adhesive Petri dish in RPMI1640 supplemented with FBS (10%), L-glutamine (2 mM), penicillin (100 U/ml) and streptomycin (100 µg/ml), at 37 °C, 5% CO_2_, 95% humidity. After incubation, medium containing non-adherent cells was gently removed and the plate with adherent cells was put on ice for 30 minutes. Afterwards, monocyte layer was harvested using a scraper.

Non-adherent cells were placed in a separated plate and cultured in RPMI1640 with FBS (10%), L-glutamine (2 mM), penicillin (100 U/ml) and streptomycin (100 µg/ml) and IL-2 (5 ng/ml) and used as effector cells for co-culture with DCs.

A total of 1 × 10^6^ adherent cells (comprising mostly monocytes)/1 ml were placed on 24-well plates in medium supplemented with GM-CSF (50 ng/ml) and IL-4 (100 ng/ml) for 7 days. On day 3 half of the medium was replaced with fresh medium containing cytokines. On day 6, cells were subjected to maturation for 24 hours in the presence of LPS (50 µg/ml), cancer cell lysates, TCM or mixtures of these. Lysates and TCM were obtained from the culture of HCT116 and HT29 cell lines (allogenic in relation to DCs).

Cell differentiation and maturation were monitored and documented with the use of Olympus CKX53 inverted microscope coupled with digital camera Olympus SC50 (Olympus, Japan). The analysis and measurements of cells sizes were performed with the use of Olympus cellSens Software

Some cancer cells were counted and afterwards directed to the lysate preparation. Lysates were prepared by 4 repeating freeze-thaw cycles (by the sequential keeping vials with cells in −80 °C and + 36 °C) followed by filtration through 0.2 µm strainer. DCs were stimulated with lysates and the proportion between the number of cancer cells taken for lysates preparation and DCs was 1:1.

### Magnetic isolation of monocytes from CRC patients blood and DCs expansion

3–6 ml of peripheral blood was collected from CRC patients before surgical resection of CRC. CD14^+^ monocytes were positively selected using anti-CD14 antibody conjugated with magnetic microbeads (EasySep™ Human CD14 Positive Selection Kit, StemCell Technology) according to the manufacturer’s instruction. Briefly, cells were incubated with anti-CD14 tetrameric antibody complexes and dextran-coated magnetic particles. Labeled cells were separated using an EasySep™ magnet. Stained cells remained in tube while CD14^−^ cells were poured off to another tubes and used as effector cells during co-culture with DCs.

A total of 2 × 10^5^ monocytes/200 µl were placed on 96-well plates and expanded according to the same protocol as we described in previous paragraph for peripheral blood monocyte–derived DCs. The lysates and TCM used for DC stimulation were obtained from the autologous cancer cells isolated from tumor fragments of CRC patients.

### Flow cytometric analysis of cells phenotype

CRC lines, cells separated from human CRC tissue fragments, DCs and effector leukocytes (mentioned in previous paragraphs) were stained with the following cocktail of monoclonal antibodies purchased from BD Biosciences, USA: anti-CD29-APC (clone MAR4, IgG1κ), anti-CD44-FITC (clone C26, IgG2bκ), anti-CD95-PE (clone DX2, C3H/Bi IgG1κ), anti-FasL Biotin (clone NOK-1, IgG1) coupled with Streptavidin-APC, anti-CD3-PE (clone UCHT1, IgG1κ), anti-CD4-FITC (clone PA-T4, IgG1κ), anti-CD11c-APC (clone S-HCL-3, IgG2b),anti-CD14-PerCP (clone MøP9, IgG2b), anti-CD25-PE (clone M-A251, IgG1κ), anti-CD56 (clone B159, IgG1κ), anti-CD80-PE (clone L307, IgG1κ), anti-CD83-APC (clone HB15e, IgG1κ), anti-HLA-DR-PerCP (clone L243, IgG2a). Anti-CD133/2-PE (clone 293C3, IgG2bκ) monoclonal antibodies were purchased from MiltenyiBiotec. After 30 min incubation in the dark, samples were fixed with 1% PFA or PBS + 1 mM EDTA for adherent or spherical cells, respectively, and prepared for further analysis. Flow cytometric analysis was performed using FACS Calibur flow cytometer (BD Biosciences, USA) with BD CellQuest Pro Software.

### Measurement of endocytotic activity of dendritic cells

Endocytotic activity of 1 × 10^5^ cells/ 100 µl/ well DCs was assessed by adding FITC-dextran (1 mg/ml, Merck, Poland) to the culture medium for 2 h at 37 °C (control on ice). Thereafter, cells were extensively washed, and the uptake of FITC-dextran was analyzed by flow cytometry (FACS Calibur flow cytometer, BD).

### Measurements of cytokine concentrations

The levels of soluble IL-10, IL-12p70, IFNγ, FasL and granzymes were analyzed in supernatants from cultures of *in vitro* modified DCs derived from both peripheral blood monocytes and CRC patient blood – monocytes using BD Cytometric Bead Array Flex Set System kits (BD Biosciences, USA) according to the manufacturer’s instruction. Briefly, Capture Beads were transferred into all tubes and samples were incubated for 1 h at RT. Afterwards, PE Detection Reagents were added to each tube, which than were incubated next 2 h at RT. Captured beads, detection reagents (reporter antibodies) and samples were incubated together to form sandwich complexes. After double washing the samples were resuspended in washing buffer and analyzed in FACS Calibur flow cytometer (BD Biosciences, USA). Fluorescence intensity was proportional to the amount of a given cytokine in a vial and estimated according to the standard curves acquired after analysis of standard dilutions. Data from cytometric analysis were transformed into graphical and tabular formats using FCAP Array Software. Finally, results were presented as pg/ml.

### Measurement of cancer cell – killing activity of stimulated DCs

Modified peripheral blood monocyte–derived DCs were co-cultured with HCT116 and HT29 CRC cell lines (allogenic co-culture) for 24 h. Unstimulated immature DCs (iDC) served as a negative control. Following the incubation cells were harvested and used for viability assays. The experiments were performed in triplicate.

### Viability assay

The 7-aminoactinomycin D (7-AAD) dye (BD Biosciences, USA) was used for death cell evaluation. After adding 10 µm of 7AAD and anti-CD133 antibodies our samples were incubated for 30 minutes, washed and resuspended in PBS prior to cytometric analysis FACS Calibur flow cytometer (BD Biosciences, USA). Anti-CD133 antibodies were used to confirm that the appropriate population of CSCs was analyzed.

### Co-culture of DCs with effector cells

Nonadherent PBMCs from peripheral blood leukocyte-platelets coats and CD14^−^ cells saved after magnetic isolation of CRC patients blood monocytes were co-cultured with *in vitro* modified DCs for 24 h. iDCs served as a negative control. Following the incubation cells were harvested and the changes of T regulatory-like lymphocytes (CD4^+^CD25^+^) and NKT cells (CD3^+^CD56^+^) proportions were evaluated with the using of FACS Calibur flow cytometer (BD Biosciences, USA). The experiments were performed in triplicate.

### Proliferation assay

The *in vitro* modified DCs and specific effector cells (mentioned above) were put in triplicate on 96-well plate in different proportions, ranging from 0.0625 to 2 (DC/effector cell number) and incubated for 24 h. After that time, cell proliferation was assessed by colorimetric CellTilter 96® Aqueous One Solution Cell Proliferation Assay (Promega Corporation), according to the manufacturer’s instructions. Briefly, 20 μl of CellTiter 96®AqueousOne Solution Reagent were added into each well of the 96-well assay plate containing the samples in 200 μl of culture medium. After 4 h of incubation the absorbance at 490 nm was recorded using a microplate reader (BioTek, Winooski, USA).

### Statistical analysis

All data obtained during the study were analyzed with the use of GraphPad Prism ver. 6.05 (GraphPad Software, San Diego, CA, USA) and the software Statistica 12 (Statsoft, Poland) according to some non-parametric tests: U Mann-Whitney, Kruskal-Wallis ANOVA, Spearman’s rank correlation coefficient. Values of *p* < *0*.*05* were considered as statistically significant.

## Electronic supplementary material

Supplementary data

## Data Availability

The datasets generated during and/or analyzed during the current study are available from the corresponding author on reasonable request.
