# Supplementary material for: Cancer stem cells as targets for DC-based immunotherapy of colorectal cancer
Source: Sci Rep. 2018 Aug 13;8:12042. doi: 10.1038/s41598-018-30525-3 (PMC6089981; doi:10.1038/s41598-018-30525-3)
Supplement: Supplementary file 1 — Supplementary data [file 41598_2018_30525_MOESM1_ESM.docx]

**Supplementary data:**

**Cancer stem cells as targets for DC-based immunotherapy of colorectal cancer**

**Authors: Magdalena Szaryńska^1*^, Agata Olejniczak^1^, Jarosław Kobiela^2^, Dariusz Łaski^2^, Zbigniew Śledziński^2^, Zbigniew Kmieć^1^**

^1^Department of Histology, Medical University of Gdansk, 80-210 Gdansk, Poland

^2^Department of General, Endocrine and Transplant Surgery, Medical University of Gdansk, 80-214 Gdansk, Poland

|  | **Age**  **[years]** | **Sex** | **TNM status** | **Local lymph nodes metastases** | **Distant metastases** | **Localization of tumor** |
| --- | --- | --- | --- | --- | --- | --- |
| **1.** | 88 | Female | pT III | N 0 | M 0 | Colic flexures |
| **2.** | 74 | Female | pT III | N 0 | M 0 | Ascending colon |
| **3.** | 75 | Female | pT IVa | N IIb | M 0 | Cecum |
| **4.** | 71 | Male | pT IVa | N Ib | M 0 | Sigmoid colon |
| **5.** | 81 | Male | pT III | N 0 | M 0 | Sigmoid colon, rectum |

Supplementary table S1. Characteristics of CRC patients included into the study which represent the group of donors which CSCs survived in culture for extended passages.


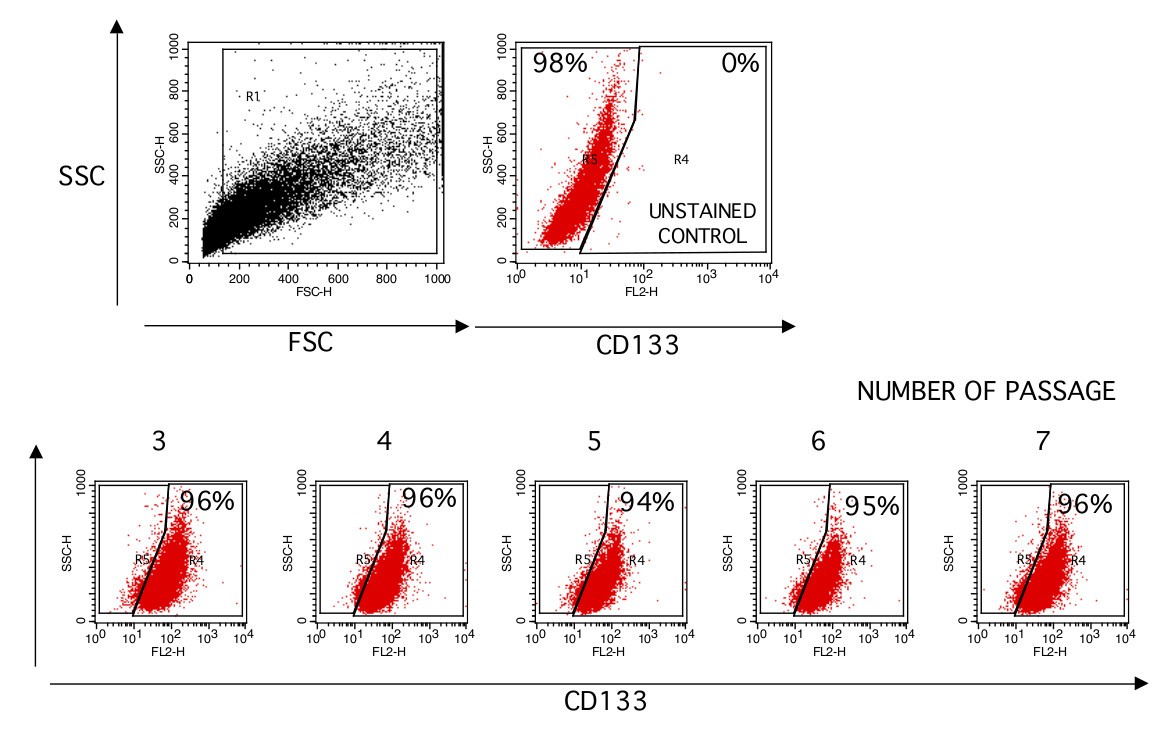


Supplementary figure S1. Exemplary cytometric analysis of CSCs along the expansion. Data are derived from the analysis of cells from third to seventh passage. First the small cells and debris were excluded (SSC/FSC dot plot). Next, CD133-negative and CD133-positive populations were distinguished and compared at different time points.


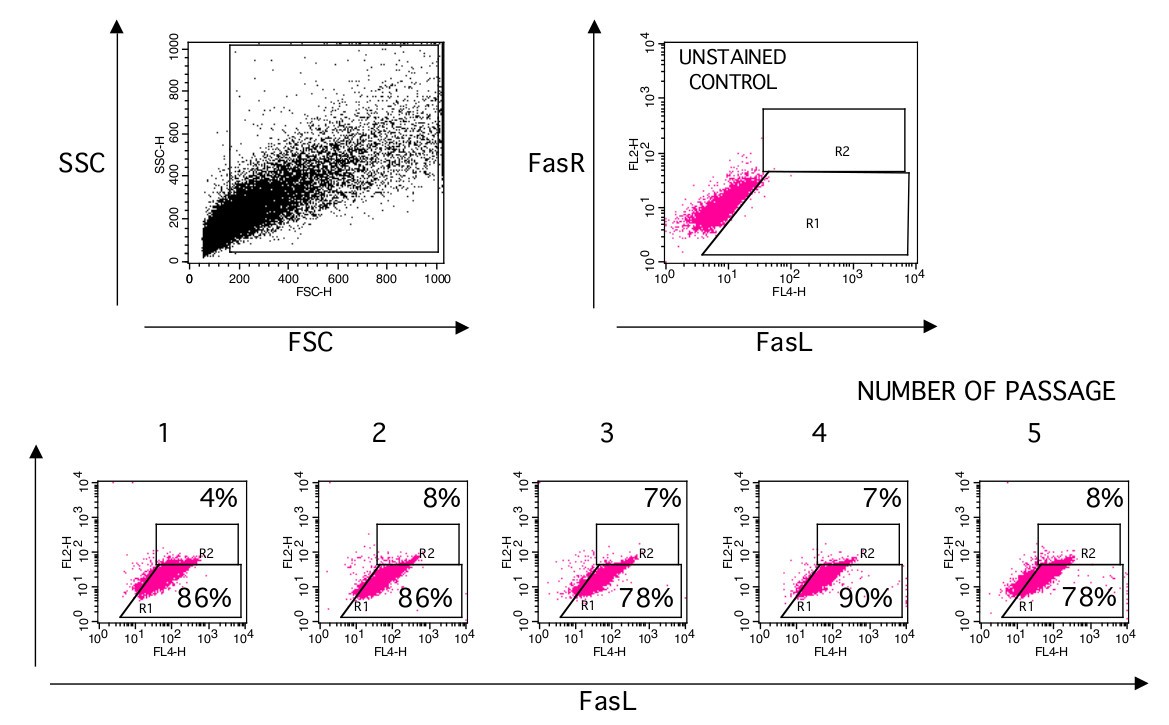


Supplementary figure S2. Exemplary cytometric analysis of CSCs along the expansion. Data are derived from the analysis of cells from third to seventh passage. First the small cells and debris were excluded (SSC/FSC dot plot). Next, FasR^+^FasL^+^ cells were identified and compared at different time points.


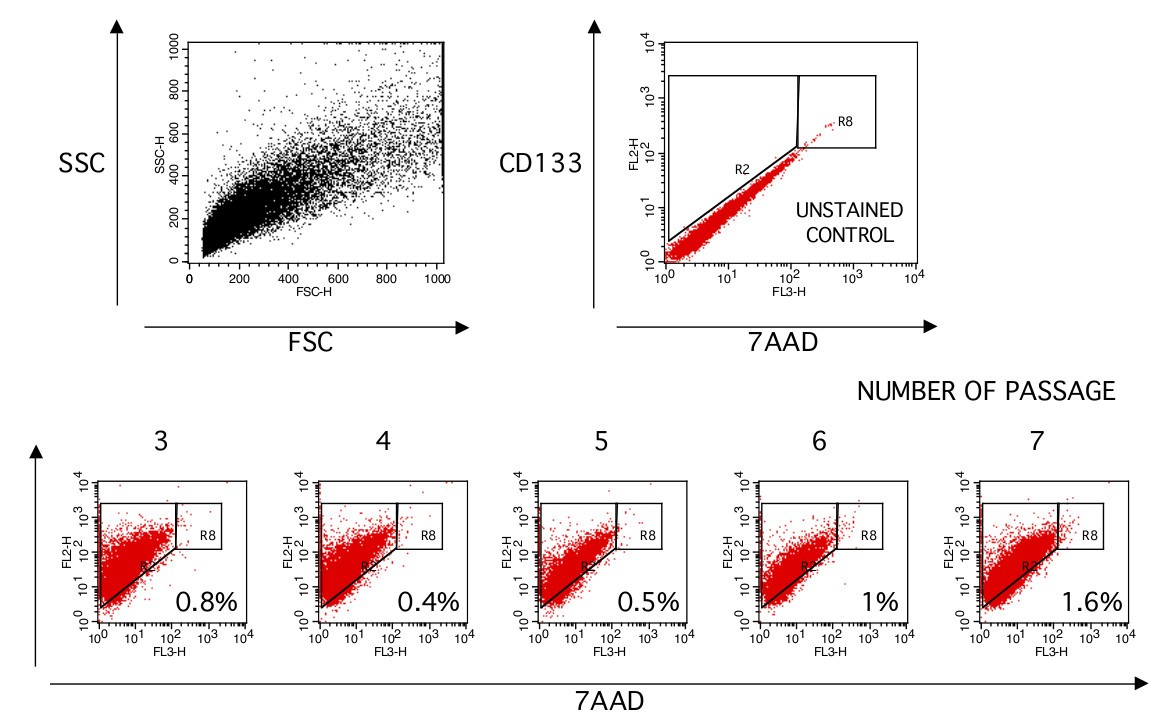


Supplementary figure S3. Exemplary cytometric analysis of CSCs’ viability along the expansion. Data are derived from the analysis of cells from third to seventh passage. First the small cells and debris were excluded (SSC/FSC dot plot). Next, cells presenting fluorescence from accumulated 7AAD dye were counted. We compared the proportion of 7AAD^+^ cells at different time points.


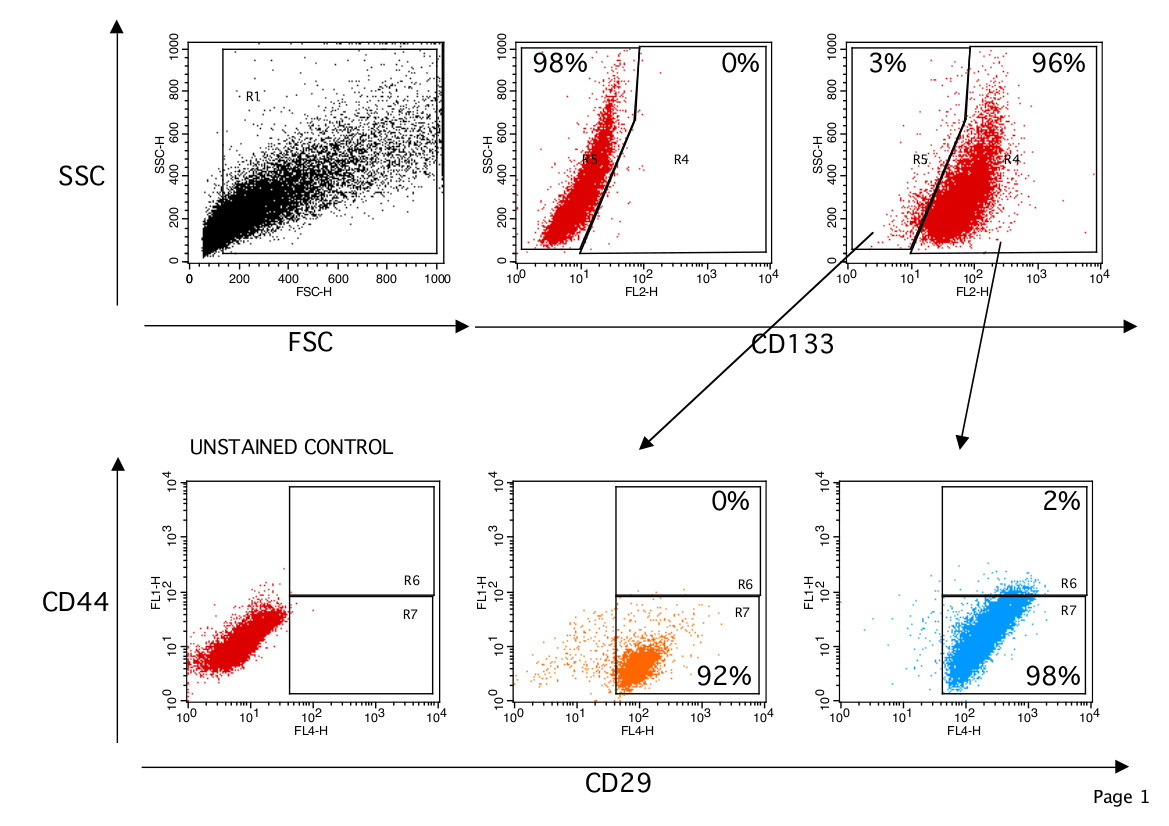


Supplementary figure S4. Exemplary gating strategy for CSCs acquired from fragments of human colorectal cancer tissue. First, the small cells and debris were excluded (SSC/FSC dot plot). Next, CD133-negative and CD133-positive populations were distinguished. Finally, CSCs were identified using anti-CD29 and anti-CD44 monoclonal antibodies in order to characterize detailed phenotype of cancer cells postulated to be CSCs. The inset numbers represent the percentage of the gated cells in the respective gating step.


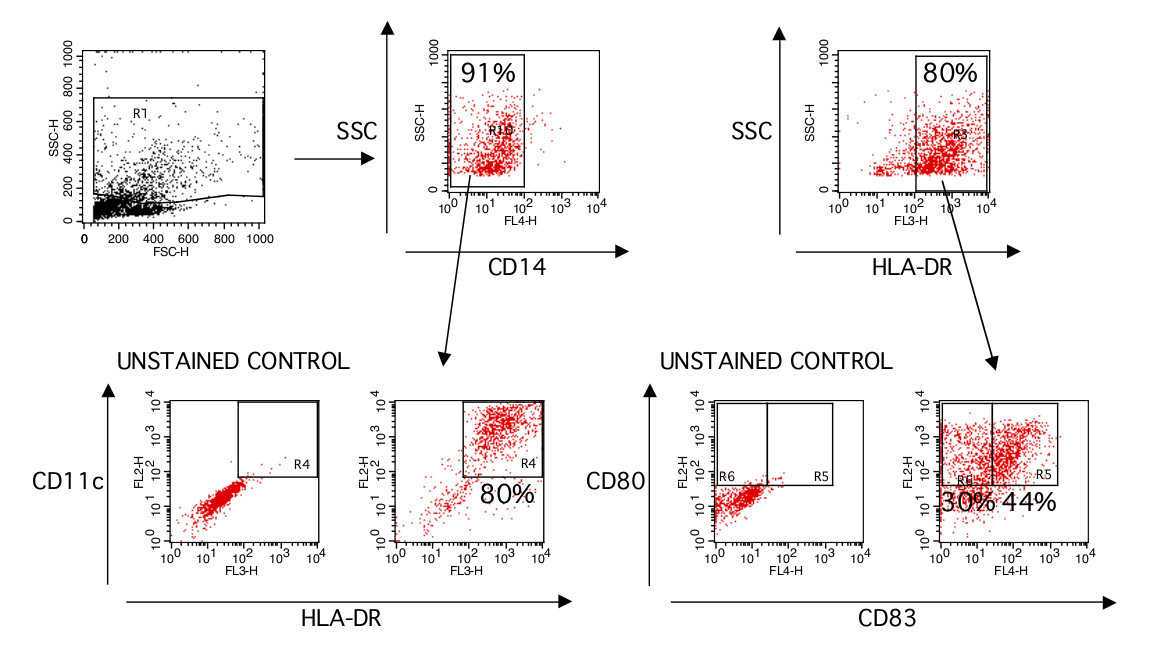


Supplementary figure S5. Exemplary gating strategy for human DCs isolated from blood of CRC patients. First the small cells were excluded (SSC/FSC dot plot). Next, CD14 cells were eliminated from further analysis (proportion of these cells was always <0.5%). Expression of maturation markers on CD14^-^ cells was determined using anti-CD11c and anti-HLA-DR monoclonal antibodies. The second mix of antibodies allowed to distinguish activated DCs which were defined as HLA-DR^+^CD80^+^CD83^+^. The inset numbers represent the percentage of the gated cells in the respective gating step.
